# CircTRIM1 encodes TRIM1-269aa to promote chemoresistance and metastasis of TNBC via enhancing CaM-dependent MARCKS translocation and PI3K/AKT/mTOR activation

**DOI:** 10.1186/s12943-024-02019-6

**Published:** 2024-05-16

**Authors:** Yaming Li, Zekun Wang, Jingwen Yang, Yuhan Sun, Yinqiao He, Yuping Wang, Xi Chen, Yiran Liang, Ning Zhang, Xiaolong Wang, Wenjing Zhao, Guohong Hu, Qifeng Yang

**Affiliations:** 1 Department of Breast Surgery, General Surgery, Qilu Hospital of Shandong University, Jinan, Shandong 250012 China; 2 School of Basic Medicine, Jining Medical College, Jining, Shandong 272067 China; 3 Pathology Tissue Bank, Qilu Hospital of Shandong University, Jinan, Shandong 250012 China; 4 Shanghai Institute of Nutrition and Health, University of Chinese Academy of Sciences, Chinese Academy of Sciences, Shanghai, 200031 China; 5 Research Institute of Breast Cancer, Shandong University, Jinan, Shandong 250012 China

**Keywords:** TNBC, circTRIM1, TRIM1-269aa, MARCKS, PI3K/AKT/mTOR

## Abstract

Peptides and proteins encoded by noncanonical open reading frames (ORFs) of circRNAs have recently been recognized to play important roles in disease progression, but the biological functions and mechanisms of these peptides and proteins are largely unknown. Here, we identified a potential coding circular RNA, circTRIM1, that was upregulated in doxorubicin-resistant TNBC cells by intersecting transcriptome and translatome RNA-seq data, and its expression was correlated with clinicopathological characteristics and poor prognosis in patients with TNBC. CircTRIM1 possesses a functional IRES element along with an 810 nt ORF that can be translated into a novel endogenously expressed protein termed TRIM1-269aa. Functionally, we demonstrated that TRIM1-269aa, which is involved in the biological functions of circTRIM1, promoted chemoresistance and metastasis in TNBC cells both in vitro and in vivo. In addition, we found that TRIM1-269aa can be packaged into exosomes and transmitted between TNBC cells. Mechanistically, TRIM1-269aa enhanced the interaction between MARCKS and calmodulin, thus promoting the calmodulin-dependent translocation of MARCKS, which further initiated the activation of the PI3K/AKT/mTOR pathway. Overall, circTRIM1, which encodes TRIM1-269aa, promoted TNBC chemoresistance and metastasis by enhancing MARCKS translocation and PI3K/AKT/mTOR activation. Our investigation has yielded novel insights into the roles of protein-coding circRNAs and supported circTRIM1/TRIM1-269aa as a novel promising prognostic and therapeutic target for patients with TNBC.

**Supplementary Information:**

The online version contains supplementary material available at 10.1186/s12943-024-02019-6.

## Introduction

Breast cancer (BC) is a serious disease that poses a significant threat to women’s health, with numerous women succumbing to it annually on a global scale. According to the latest report from the International Agency for Research on Cancer (IARC), the global incidence of new cases of breast cancer increased to 2.26 million in 2020; this number officially surpassed that of lung cancer for the first time, and breast cancer has become the most prevalent malignancy worldwide [1]. Breast cancer exhibits significant heterogeneity, with approximately 15–20% of patients with breast cancer being diagnosed with the triple-negative subtype (TNBC), which is characterized by the absence of estrogen receptor (ER), progesterone receptor (PR), and human epidermal growth factor receptor type 2 (Her-2) gene expression [2]. Our previous findings indicated that patients diagnosed with TNBC experience relatively poorer outcomes due to factors such as early metastasis, rapid proliferation, and a deficiency of molecular targets for effective treatment [3]. While surgical intervention and radiotherapy are commonly employed for patients with TNBC, adjuvant chemotherapy appears to be particularly crucial in addressing the absence of molecular targets, thereby becoming the sole systemic treatment option [4].

Compared with non-TNBC subtypes, TNBC subtypes are more sensitive to chemotherapy, particularly to cytotoxic agents such as anthracyclines and taxanes [5]. A real-world study demonstrated that chemotherapy significantly improved the overall survival rate (adjusted HR = 0.58, 95% CI = 0.46–0.73) and breast cancer-specific survival rate (adjusted HR = 0.65, 95% CI = 0.48–0.89) among patients with TNBC during the 8.2-year median follow-up [6, 7]. However, it is worth noting that TNBC cells are more likely to develop chemoresistance than other types of breast cancer, and the majority of patients with TNBC eventually develop chemoresistance. The acquisition of chemoresistance is a complex process initiated by multiple mechanisms, including reduced cell apoptosis and the regulation of autophagy [8]. Once chemoresistance occurs, cancer cells become more aggressive and prone to metastasis [9], which are the most prevalent factors contributing to treatment failure, disease recurrence, and eventual mortality in clinical patients [10]. Therefore, there is an urgent need to elucidate the molecular mechanisms and identify novel targets for TNBC chemoresistance and metastasis.

The advancement of next-generation sequencing technologies has facilitated the identification of a growing number of previously undiscovered transcripts. Circular RNA (circRNA) is a newly recognized category of endogenous RNA transcript that possesses a covalently closed loop structure due to the nonsequential back-splicing of pre-mRNA transcripts, which was initially misinterpreted as a byproduct of splicing errors [11]. Unlike linear RNAs, circRNAs do not possess 5’ caps or 3’ tails and exhibit distinct characteristics, such as longer half-lives, greater evolutionary conservation, and enhanced resistance to RNase R digestion [12]. Although most previous studies have focused on the noncoding roles of circRNAs, it is worth noting that most circRNAs arise from the coding exons of host genes, and a majority of them contain translatable open reading frames (ORFs). Moreover, circRNAs are mainly localized in the cytoplasm, where they can bind to ribosomes and initiate translation, as most circRNAs contain an IRES [13]. For instance, circPPP1R12A can promote colon cancer pathogenesis and metastasis by activating the Hippo–YAP signaling pathway [14]. Circ-AKT3 inhibited the proliferation, radioresistance and in vivo tumorigenicity of glioblastoma cells by inhibiting the phosphorylation of AKT Thr308 [15]. We have also reported that circ-EIF6 encodes EIF6-224 aa to facilitate TNBC proliferation and metastasis by activating the Wnt/beta-catenin pathway [16]. However, the functions and underlying mechanisms of protein-coding circRNAs in TNBC chemoresistance and progression remain largely unexplored, and identifying novel protein-coding circRNAs that play oncogenic roles in TNBC and elucidating the underlying mechanisms might provide novel therapeutic targets for TNBC treatment.

In the present study, we identified an unpublished circRNA, hsa_circ_0002153 (also termed circTRIM1), that was upregulated in DOX-resistant TNBC cells and tissues by transcriptome and translatome RNA-seq. The expression of circTRIM1 was also associated with clinicopathological characteristics and poor prognosis in patients with TNBC. Further experiments demonstrated that circTRIM1 could be translated into a novel protein termed TRIM1-269aa in an IRES-dependent manner. In vitro and in vivo studies demonstrated that circTRIM1 promoted the chemoresistance and metastasis of TNBC cells by encoding TRIM1-269aa. Moreover, TRIM1-269aa could be encapsulated within exosomes, thereby exerting its functions through exosome transmission. Mechanistically, TRIM1-269aa enhanced the interaction between MARCKS and calmodulin, which further facilitated the calmodulin-dependent translocation of MARCKS. Translocated MARCKS could further activate the downstream PI3K/AKT/mTOR pathway, leading to the malignant behaviors of TNBC cells. In conclusion, our investigation revealed that circTRIM1 encoded TRIM1-269aa to promote the chemoresistance and metastasis of TNBC by enhancing calmodulin-dependent MARCKS translocation and PI3K/AKT/mTOR activation.

## Results

### CircTRIM1 is a potential coding circRNA that is upregulated in DOX-resistant TNBC cells and is correlated with poor prognosis in patients with TNBC

**Fig. 1:**
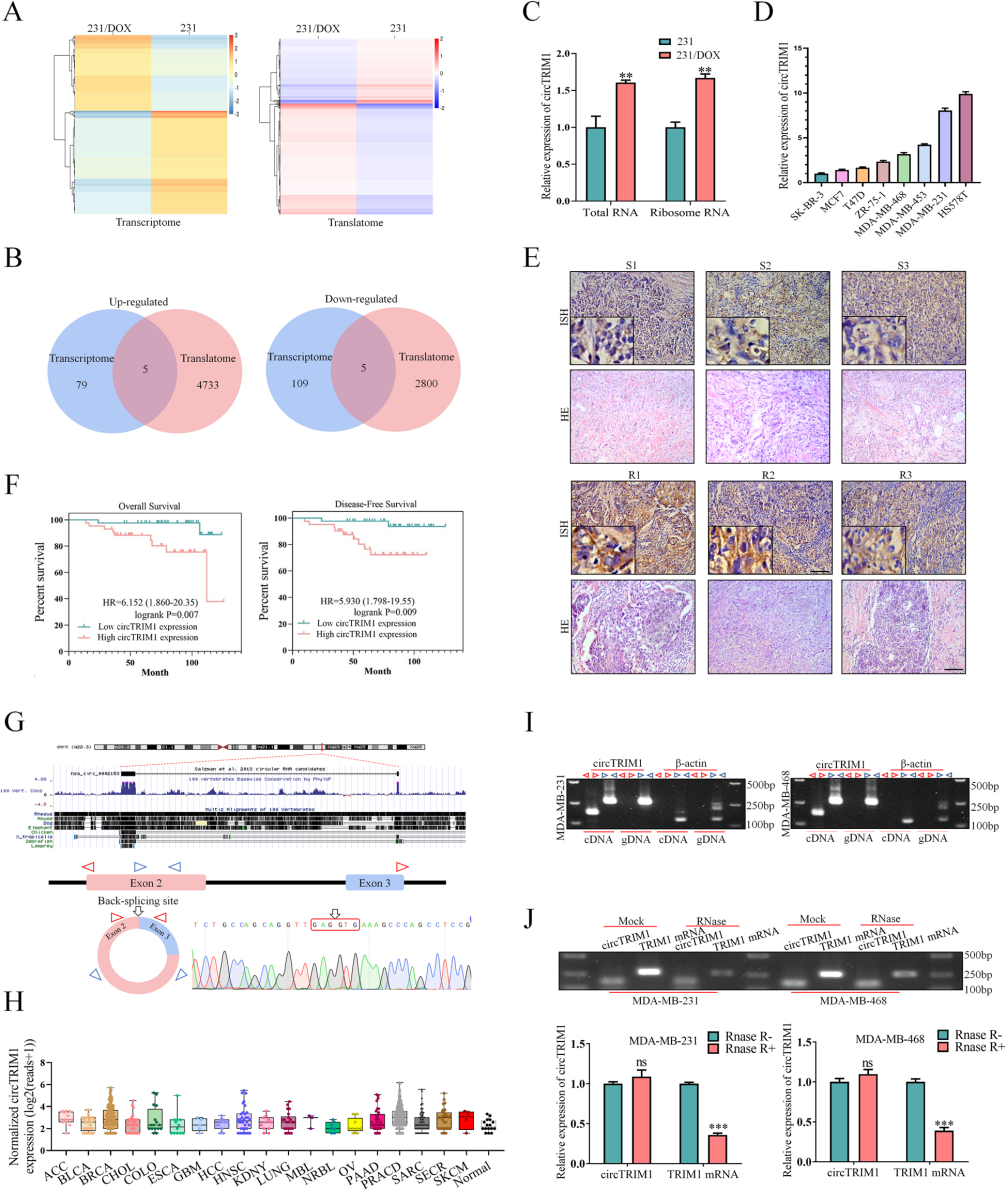
CircTRIM1 is an upregulated circRNA in DOX-resistant TNBC with translational and prognostic potential.** A** Transcriptome sequencing (left panel) and translatome sequencing (right panel) of MDA-MB-231 and 231/DOX (DOX-resistant subcell line) cells. **B** Dysregulated circRNAs identified by both transcriptome sequencing and translatome sequencing. **C** The relative expression of circTRIM1 in 231 and 231/DOX cell lines was examined by qRT‒PCR. **D** Expression of circTRIM1 in breast cancer cell lines with different hormone receptor statuses. **E** ISH of circTRIM1 in primary and metastatic TNBC tissues. Scale bars = 100 μm. **F** Tissues were collected from 86 patients with TNBC, and survival analyses were performed to evaluate the association between circTRIM1 expression and overall or recurrence-free survival (*n* = 43 patients in each group). **G** Upper panel: the schematic diagram indicates the genomic loci of circTRIM1. Lower panel: divergent (red) and convergent (blue) primers for circTRIM1 used in this study, and Sanger sequencing conducted following PCR using the indicated divergent flanking primers confirmed the “head-to-tail” splicing of circTRIM1 in TNBC cells. **H** Expression of circTRIM1 in tumors from the MioncoCirc database. **I** PCR assays with divergent or convergent primers indicated the presence of circTRIM1 in cDNA but not in gDNA. Actin was used as a negative control. **J** Total RNA extracted from TNBC cells was treated with or without RNase R, and the expression of circTRIM1 or TRIM1 mRNA was measured by qRT‒PCR. ns, nonsignificant; **P < 0.01; ***P < 0.001

To identify potential functional circRNAs that are correlated with DOX resistance in TNBC, we first performed circRNA-seq (transcriptome sequencing) in both MDA-MB-231 and 231/DOX (doxorubicin-resistant) cells. As shown in Fig. 1A, In the left panel, 84 circRNAs were upregulated and 114 circRNAs were downregulated in 231/DOX cells, and the expression levels, lengths, and types of detected circRNAs are also presented (Figure S1.A, upper panel). To further explore circRNAs with translation potential, RNC-seq (i.e., translatome sequencing) was then performed to filter differentially expressed circRNAs that were combined with ribosomes in cells, and 4,778 upregulated and 2,805 downregulated circRNAs were identified in the 231/DOX group (Fig. 1A, right panel; Figure S1.A, lower panel). By intersecting the differentially expressed circRNAs identified in both the transcriptome and translatome sequencing results mentioned above, we identified 10 circRNAs that were dysregulated in the 231/DOX cells (Fig. 1B). The basic information and potential coding ORFs of the 10 circRNAs from the circBank database [17] were also obtained (Figure S1.B).

We further examined the expression profiles of the 10 circRNAs in the MDA-MB-231 and 231/DOX cell lines by qRT‒PCR and confirmed that circTRIM1 was upregulated in both the total and ribosomal RNA of 231/DOX cells (Fig. 1C). Moreover, circTRIM1 was shown to be overexpressed in TNBC cell lines compared with non-TNBC cell lines (Fig. 1D). An ISH assay also verified the significant upregulation of circTRIM1 in chemoresistant TNBC tissues compared with chemosensitive TNBC tissues (Fig. 1E). Next, to evaluate the prognostic significance of circTRIM1, 86 TNBC tissues were selected and equally divided into two groups based on circTRIM1 expression measured by qRT‒PCR. As shown in Fig. 1F, survival analyses demonstrated that high circTRIM1 expression was correlated with poor overall survival (OS) and disease-free survival (DFS). The associations between circTRIM1 expression and the clinicopathological characteristics of patients with TNBC are shown in Table 1 and indicated circTRIM1 expression was closely correlated with recurrence. Univariate and multivariate analyses were also conducted, which demonstrated that circTRIM1 expression was an independent prognostic factor for both OS (Table 2) and DFS (Table 3). Building upon the aforementioned results, circTRIM1 was ultimately selected as a potential functional coding circRNA involved in DOX resistance in TNBC.

**Table 1:** Association between Clinicopathological variables and circTRIM1 expression in TNBC patients

		circTRIM1 expressions		
Variable	Cases (*n* = 86)	Low	High	*P* value
**Age**				
≤ 45	23	13	10	0.465
> 45	63	30	33	
**Histologic subtypes**				
IDC	80	38	42	0.202
non-IDC	6	5	1	
**Histologic grade**				0.664
G2	40	18	22	
G3	41	22	19	
Unknown	5	3	2	
**Tumor size**				0.262
≤ 2	29	12	17	
> 2	55	29	26	
Unknown	2	2	0	
**Lymph node status**				0.117
Negative	55	31	24	
Positive	31	12	19	
**Ki67 status**				0.078
Low	9	7	2	
High	77	36	41	
**Recurrence**				**0.049**
No	75	41	34	
Yes	11	2	9	

**Table 2:** Univariate and multivariate analyses of prognostic factors (OS) for patients with TNBC

Variable	Univariate analysis (OS)		Multivariate analysis (OS)	
	HR (95% CI)	*P* value	HR (95% CI)	*P* value
**Age**				
Age ≤ 45	Reference	-		
Age > 45	0.892 (0.234–3.398)	0.867		
**Histological type**				
IDC	Reference	-		
non-IDC	0.043 (0.000-446.278)	0.505		
**Histological grade**				
G2	Reference	-		
G3	1.893 (0.553–6.482)	0.310		
Unknown	-	-		
**Tumor size**				
≤ 2 cm	Reference	-		
> 2 cm	1.274 (0.361–4.495)	0.706		
Unknown	-	-		
**Lymph node status**				
Negative	Reference	-	Reference	-
Positive	**3.777 (1.100-12.964)**	**0.035**	**3.871 (1.084–13.819)**	**0.037**
**Ki67 status**				
Low		-		
High	1.254 (0.160–9.849)	0.829		
**circTRIM1 expression**				
Low	Reference	-	Reference	-
High	**6.544 (1.384–30.942)**	**0.018**	**6.884 (1.389–34.119)**	**0.018**

**Table 3:** Univariate analysis of prognostic factors (DFS) for patients with TNBC

Variable	Univariate analysis (DFS)	
	HR (95% CI)	*P* value
**Age**		
≤ 45	Reference	-
Age > 45	1.759 (0.380–8.148)	0.470
**Histologic subtypes**		
IDC	Reference	-
non-IDC	0.043 (0.000-537.755)	0.514
**Histological grade**		
G2	Reference	-
G3	0.919 (0.281–3.013)	0.890
Unknown	-	-
**Tumor size**		
≤ 2 cm	Reference	-
> 2 cm	1.554 (0.412–5.861)	0.515
Unknown	-	-
**Lymph node status**		
Negative	Reference	-
Positive	2.774 (0.844–9.119)	0.093
**Ki67 status**		
Low	Reference	-
High	0.627 (0.135–2.907)	0.551
**circTRIM1 expression**		
Low	Reference	-
High	**5.615 (1.208–26.094)**	**0.028**

### Characteristics of circTRIM1 in TNBC

Based on the USCS Genome Browser [18], circTRIM1 arose from exons 2–3 of the TRIM1 (also known as MID2) gene (chrX: 107,083,899–107,097,934) with a mature length of 812 nt. Specific divergent (red) and convergent (blue) primers for circTRIM1 were designed, and the junction sequence of circTRIM1 was verified by Sanger sequencing in TNBC cells, suggesting the endogenous existence of circTRIM1 in TNBC (Fig. 1G). The MiOncoCirc database [19] also indicated that circTRIM1 was widely expressed among tumors (Fig. 1H). cDNA and gDNA templates were then extracted from TNBC cell lines, and PCR assays revealed that circTRIM1 could only be amplified from cDNA rather than from gDNA, indicating that circTRIM1 was a backsplicing product of the pre-mRNA (Fig. 1I). Moreover, RNase R was added to the total RNA of TNBC cells, and we demonstrated that circTRIM1 was more resistant to RNase R digestion than was the linear form of TRIM1 mRNA (Fig. 1J). We also found that circTRIM1 could only be efficiently reverse transcribed with random primers, while the relative expression of circTRIM1 markedly decreased when oligo-dT primers were used, which was in accordance with the circular structure of circTRIM1 (Figure S1.C). The stability of circTRIM1 was then evaluated by an actinomycin D assay, and we found that the half-life of circTRIM1 was markedly longer than that of the linear form of TRIM1 mRNA (Figure S1.D). Taken together, our results confirmed that circTRIM1 is an endogenously expressed circRNA in TNBC.

### CircTRIM1 encodes a novel protein termed TRIM1-269aa

**Fig. 2:**
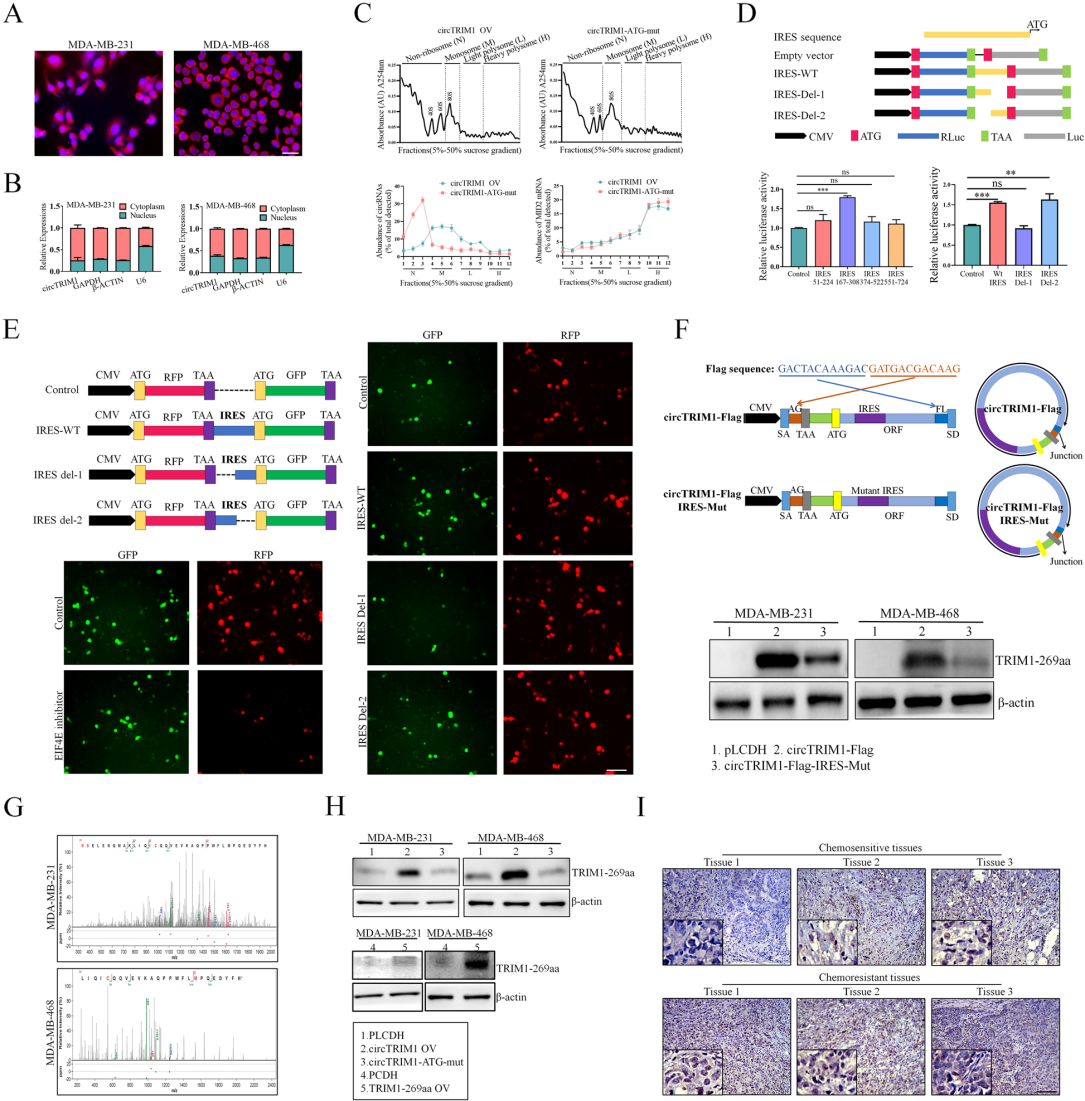
CircTRIM1 can be translated into a novel peptide termed TRIM1-269aa in an IRES-dependent manner. The subcellular locations of circTRIM1 were detected by FISH (**A**) and nucleocytoplasmic fractionation (**B**) assays. Scale bars = 40 μm. **C**. Polysome fractions (N, nonribosome; M, monosome; L, light polysome; and H, heavy polysome) were extracted from 293T cells overexpressing circTRIM1 or circTRIM1-ATG-mut (upper panel). qRT‒PCR was then performed to evaluate the ribosome-binding potential of circTRIM1 and circTRIM1-ATG-mut (lower left panel). TRIM1 mRNA was detected as a positive control (lower right panel). **D**. The full-length or truncated IRES sequences of circTRIM1 were cloned and inserted into RLuc-Luc vectors (upper panel), and the translation initiation ability of the IRES was detected by dual-luciferase reporter assays (lower panel). **E**. Upper left panel: Full-length or truncated IRES sequences were cloned between RFP and GFP as indicated to construct reporter plasmids. Lower left panel: Plasmids were transfected into 293T cells with or without eIF4E inhibitor treatment. RFP and GFP signals were detected. Right panel: Full-length or truncated IRES vectors were transfected into 293T cells, and RFP and GFP signals were detected. Scale bars = 200 μm. **F**. Upper panel: circTRIM1-Flag was designed to detect the circTRIM1-encoded peptide, and the Flag sequence was divided and cloned on both sides of the circRNA sequence; the circular junction was moved to the inside of the Flag sequence. Circularization of this vector produced the same circRNA as endogenous circTRIM1, except for the addition of a Flag tag after the ORF. A similar circTRIM1-Flag expression vector with a mutant IRES sequence was also constructed (circTRIM1-Flag-IRES-mut). Lower panel: An anti-FLAG antibody was used to detect the expression of TRIM1-269aa-Flag. **G**. LC‒MS data were downloaded from the ProteomeXchange database, and the specific sequences of TRIM1-269aa were identified in the MDA-MB-231 and MDA-MB-468 cell lines. **H**. Effects of the circTRIM1 overexpression vector (circTRIM1 OV), the circTRIM1 overexpression vector with the ATG mutant (circTRIM1-ATG-mut) and the TRIM1-269aa overexpression vector (TRIM1-269aa-Flag) on the expression of TRIM1-269aa. **I**. An IHC assay was performed to detect the expression of TRIM1-269aa in chemosensitive and chemoresistant TNBC tissues. Scale bars = 100 μm. ns, nonsignificant; **P < 0.01; ***P < 0.001

As we demonstrated that circTRIM1 is an endogenously expressed circRNA with ribosome-binding ability and an ORF region, we further investigated whether circTRIM1 could be translated into a protein. FISH and subcellular fractionation assays indicated that circTRIM1 is located mainly in the cytoplasm of TNBC cells, providing the basis for ribosome binding and further translation (Fig. 2A and B). Next, we performed a sucrose density gradient centrifugation-based polysome analysis to explore the ribosome-binding ability of circTRIM1. As shown in Fig. 2C, upper panel, circTRIM1 and circTRIM1-ATG-mut vectors were transfected into 293T cells, and the ribosome fractions were extracted and divided into nonribosome (N), monosome (M), light polysome (L), and heavy polysome (H) fractions in both cell lines. The distributions of circTRIM1 and circTRIM1-ATG-mut among the fractions were detected, and we found that circTRIM1 mainly bound to the M and L fractions, while circTRIM1-ATG-mut only bound to the N fractions, suggesting that the ATG mutation of the ORF region could interfere with the ribosome-binding ability of circTRIM1 (Fig. 2C, lower left panel). TRIM1 mRNA was also detected among the fractions as a control, and no significant change was detected after ATG mutation (Fig. 2C, lower right panel). Because the IRES element has been reported to be an important factor affecting the translation ability of circRNAs [20, 21], we then evaluated whether circTRIM1 contains an IRES element based on the CircBank and circRNADB databases [17, 22]. As shown in Figure S1.E, four IRES elements were predicted by both databases, including residues 51–224, 167–308, 374–522 and 551–724 of the circTRIM1 sequence. To investigate the translation initiation abilities of the 4 IRES elements, we first constructed an RLuc-Luc vector in which Luc expression was controlled by its preceding sequence (Fig. 2D, upper panel). The abovementioned 4 IRES elements were then inserted into the RLuc-Luc vector, and a dual-luciferase assay was performed to examine the activities of the predicted IRES elements. As shown in Fig. 2D, lower left panel, we found that the 167–308 IRES had the strongest translation initiation ability. Further dividing IRES 167–308 (IRES-WT) into IRES 167–238 (IRES-Del-1) and IRES 238–308 (IRES-Del-2) (Fig. 2D, upper panel), we found that the 449–522 IRES had a major effect on the 238–308 IRES (Fig. 2D, lower right panel). To further validate the translation initiation ability of IRES 167–308, RFP and GFP were cloned and inserted into a dual-cistron reporter construct with putative or truncated IRES elements between them (Fig. 2E, upper left panel). The vector containing full-length IRES 167–308 was first transfected into 293T cells, after which both GFP and RFP were expressed. However, after treatment with an EIF4E (eukaryotic translation initiation factor 4E) inhibitor, RFP expression was suppressed, but GFP expression did not significantly change, suggesting that IRES 167–308-driven GFP expression occurred in a 5’ cap-independent manner (Fig. 2E, lower left panel). Furthermore, the truncated IRES sequence induced lower GFP expression than did the full-length IRES sequence, indicating that IRES residues 167–308 in circTRIM1 facilitated ribosome entry and initiated protein translation (Fig. 2E, right panel).

After confirming the translation initiation ability of IRES 167–308, the sequence of circTRIM1 was further analyzed. Figure S1.F shows the annotated junction site, IRES element, start codon, and stop codon in the circTRIM1 sequence. Additionally, an 810 nt ORF region was identified, suggesting the potential translation of a novel 269-amino acid protein (TRIM1-269aa). The amino acid sequence of TRIM1-269aa was subjected to a PubMed BLAST search [23], leading to the identification of a specific 16-amino acid sequence (KAQPPWFLMPQEDYFH), which was further used as the antigen sequence of TRIM1-269aa to generate a specific antibody (Figure S1.G). To validate the translation ability of circTRIM1, a novel circTRIM1 vector containing the Flag sequence was constructed. As shown in the upper panel of Fig. 2F, the original junction of endogenous circTRIM1 is located inside the ORF. We moved the junction to the stop codon of the ORF and shifted the Flag sequence to both sides (circTRIM1-Flag) and found that the Flag tag could only be expressed when the sequence of circTRIM1-Flag was circulated. A similar vector with a mutant IRES sequence was also constructed (circTRIM1-Flag-IRES-Mut, Fig. 2F, middle panel). The mutant sequence followed the principle of synonymous mutations, ensuring that the amino acid sequence of TRIM1-269aa remained unchanged. To avoid the emergence of a novel functional IRES structure, the mutant sequence was subjected to further evaluation by using IRESfinder [24] and dual-luciferase reporter assays (Figure S1.H, I). As shown in Fig. 2F, lower panel, the expression of the Flag tag was detected, whereas mutation of the IRES sequence resulted in decreased Flag expression, providing further evidence supporting the crucial role of the IRES sequence in the translation of circTRIM1. Because circRNA translation could also be regulated by m6A modification cooperated with the initiation factor eIF4G2 and the m6A reader protein YTHDF3 [25], we further knocked down YTHDF3 expression in TNBC cells, which had no significant effect on the expression of TRIM1-269aa, further indicating that circTRIM1 translation was regulated by IRES sequences (Figure S1. J, K). We also downloaded mass spectrometry data from the ProteomeXchange [26] database (PXD026234, PXD008522), and the specific amino acid sequence of TRIM1-269aa could be found in TNBC cell lines by using pFind [27] software (Fig. 2G). For further functional studies, we constructed a circTRIM1 overexpression vector (circTRIM1 OV), a circTRIM1 overexpression vector with an ATG mutant (circTRIM1-ATG-mut) and a TRIM1-269aa linear overexpression vector (TRIM1-269aa-Flag). These constructs were transfected into both MDA-MB-231 and MDA-MB-468 cells, and the expression of TRIM1-269aa was assessed (Fig. 2H), which further validated the endogenous translation of circTRIM1 and the specificity of the TRIM1-269aa antibody. In addition, 3 chemosensitive TNBC tumors and 3 chemoresistant TNBC tumors were selected, and the expression of TRIM1-269aa was evaluated by IHC. Our results demonstrated that TRIM1-269aa was endogenously expressed in TNBC tissues and exhibited increased expression in chemoresistant tissues (Fig. 2I). Taken together, our results demonstrated that circTRIM1 could be translated into TRIM1-269aa in an IRES-dependent manner and that TRIM1-269aa was endogenously expressed in TNBC cell lines and tissues.

### circTRIM1 knockdown suppresses DOX resistance, migration and invasion in TNBC cells

To determine whether circTRIM1 was a functional coding circRNA, we initially designed 2 specific siRNAs to knock down the expression of circTRIM1 in TNBC cells, and the positions and sequences of the siRNAs are shown in Figure S2.A. The efficiency of circTRIM1 knockdown was evaluated by qRT‒PCR (Figure S2.B) and FISH (Figure S2.C) assays, which confirmed a significant reduction in circTRIM1 expression. Moreover, the knockdown of circTRIM1 resulted in a notable suppression of TRIM1-269aa expression (Figure S2.D). The mRNA expression level of the host gene TRIM1 was then examined, and we observed that knockdown of circTRIM1 did not affect TRIM1 mRNA expression, suggesting that the subsequent experimental results were not influenced by nonspecific knockdown of TRIM1 mRNA (Figure S2.E).

**Fig. 3:**
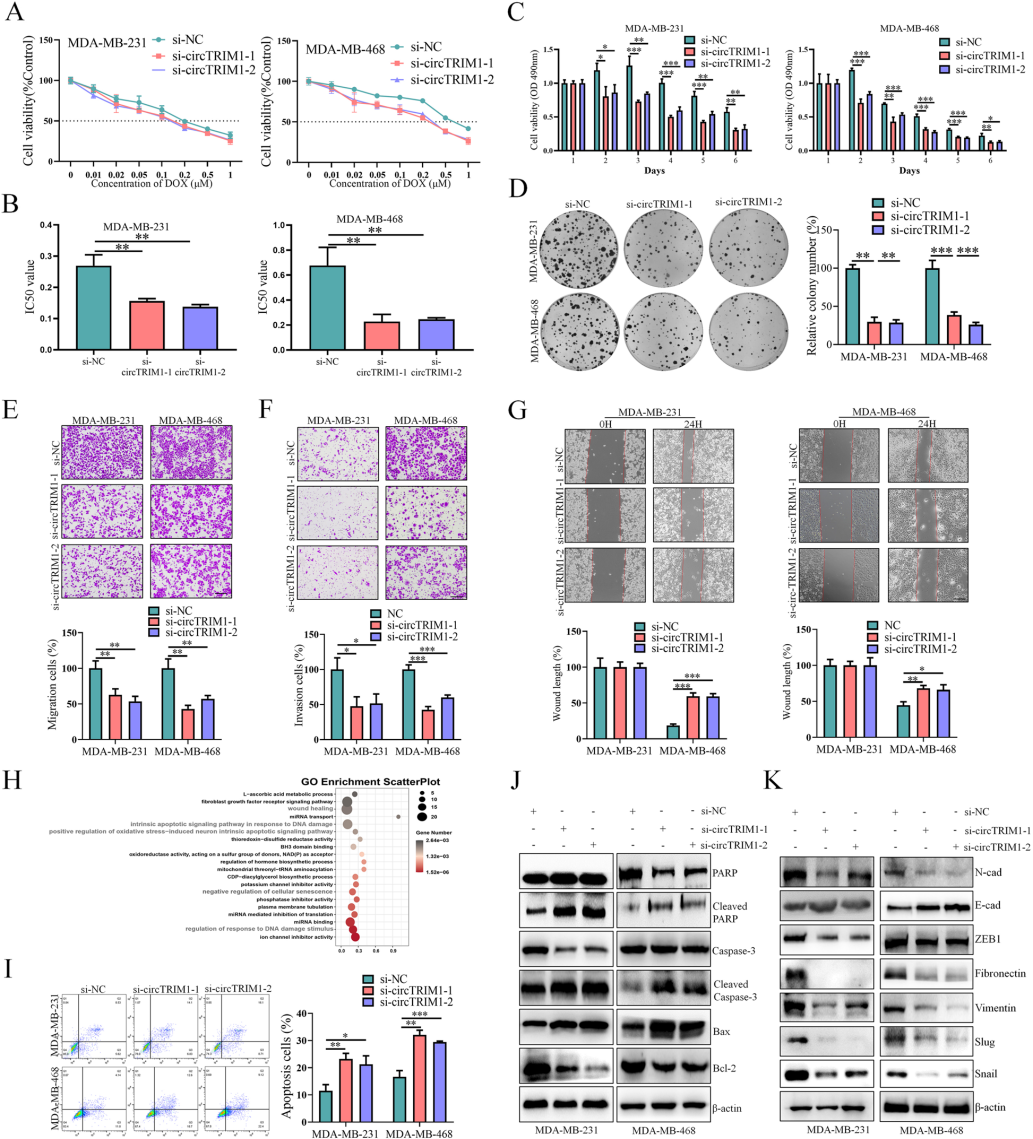
circTRIM1 knockdown suppressed the chemoresistance, migration and invasion of TNBC cells.** A** Dose-dependent cell viability after circTRIM1 knockdown was detected by MTT assay. **B** The differences in the IC50 values between circTRIM1 knockdown and control TNBC cells. **C** Time-dependent cell viability after circTRIM1 knockdown was detected by MTT assay. **D** Colony formation assays were used to determine the effect of circTRIM1 knockdown on TNBC chemoresistance. The migration (**E**) and invasion (**F**) abilities of TNBC cells with circTRIM1 knockdown were evaluated by Transwell assays. Scale bars = 200 μm. **G**. The migration abilities of TNBC cells with circTRIM1 knockdown were assessed by wound healing assays. Scale bars = 200 μm. **H**. The gene expression of TNBC cells with circTRIM1 knockdown was detected by RNA-seq, and enrichment analysis was performed to evaluate the functions of circTRIM1. **I**. Effects of circTRIM1 knockdown on DOX-induced apoptosis in TNBC cells. Effects of circTRIM1 knockdown on apoptosis-related (**J**) and EMT-related (**K**) proteins. *P < 0.05; **P < 0.01; ***P < 0.001

To verify whether circTRIM1 could influence DOX resistance, cytotoxicity assays were performed and demonstrated that downregulation of circTRIM1 increased the sensitivity of TNBC cells to DOX, as evidenced by decreased IC50 values and viability compared to those of the control group in dose-dependent (Fig. 3A and B) and time-dependent (Fig. 3C) manners. Colony formation assays also showed similar results (Fig. 3D). Previous studies reported that chemoresistant tumor cells were more likely to experience distant metastasis [28, 29]. Transwell and wound healing assays were then performed to evaluate the effects of circTRIM1 on the migration and invasion abilities of TNBC cells, which showed that knockdown of circTRIM1 led to significant suppression of the migration and invasion abilities of TNBC cells (Fig. 3E–G).

To further investigate the potential mechanisms of circTRIM1 in the malignant behaviors of TNBC, we used RNA-seq to screen the potential roles of circTRIM1 through Gene Ontology (GO) analysis in circTRIM1-knockdown MDA-MB-231 cells and found that circTRIM1 was closely associated with apoptosis, the response to DNA damage and wound healing (Fig. 3H), and we hypothesized that circTRIM1 might influence the chemoresistance of TNBC cells by promoting DOX-induced apoptosis. Based on the RNA-seq results, flow cytometry analyses were first performed to verify the effects of circTRIM1 on DOX-induced apoptosis of TNBC cells, which revealed a marked increase in the percentage of apoptotic TNBC cells following circTRIM1 knockdown (Fig. 3I). Moreover, western blotting further confirmed that the knockdown of circTRIM1 activated the apoptosis pathway in TNBC cells treated with DOX (Fig. 3J). Additionally, western blotting assays further indicated that the knockdown of circTRIM1 contributed to the inhibition of the migration and invasion abilities of TNBC cells by regulating the epithelial–mesenchymal transition (EMT) pathway (Fig. 3K).

### TRIM1-269aa, but not circTRIM1, promotes the chemoresistance, migration and invasion of TNBC cells

**Fig. 4:**
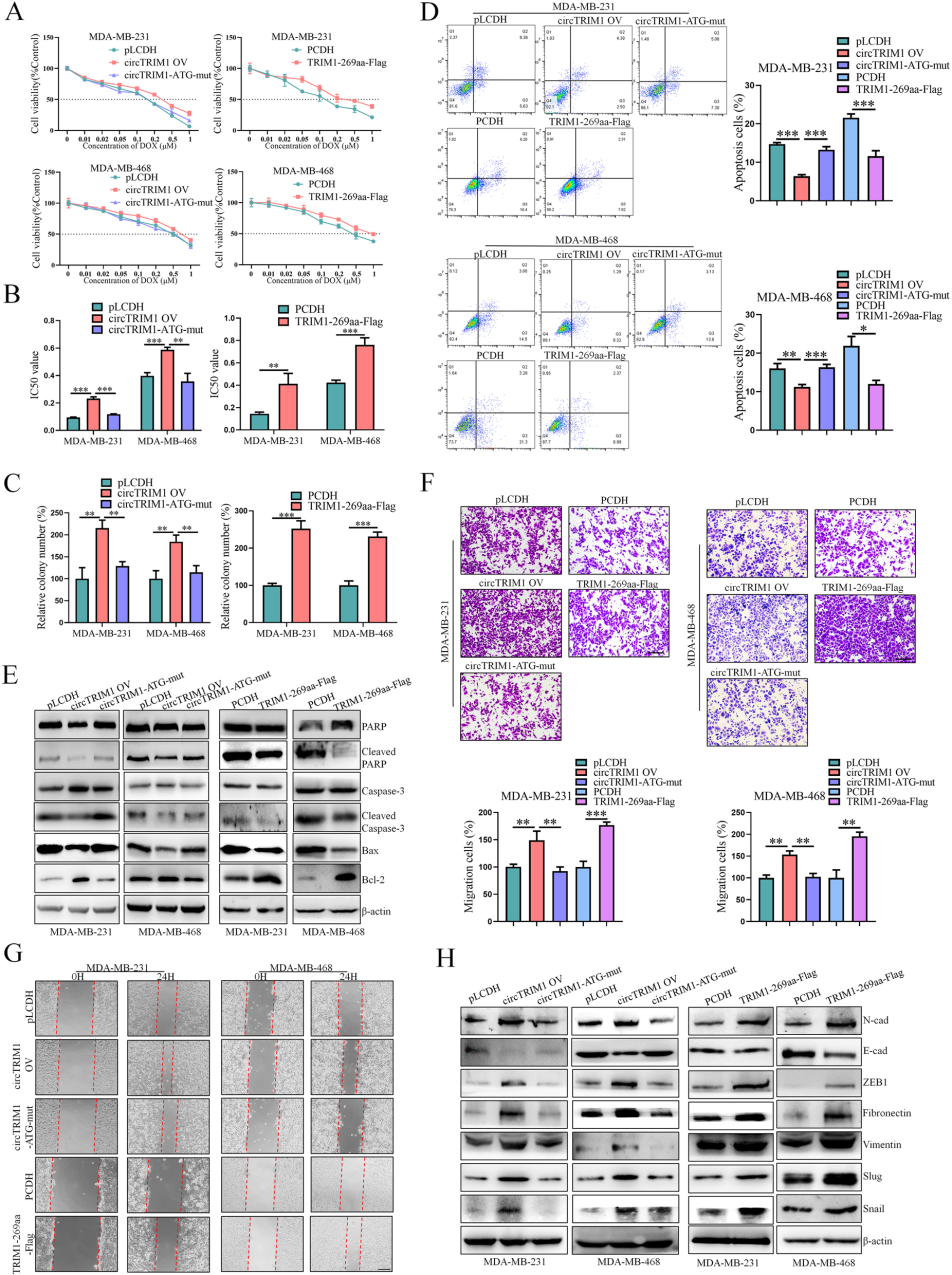
TRIM1-269aa, but not circTRIM1, promotes chemoresistance, migration and metastasis in TNBC cells.** A** Dose-dependent effects of circTRIM1, circTRIM1-ATG-mut and TRIM1-269aa on TNBC chemoresistance. **B** IC50 values of DOX in TNBC cells overexpressing circTRIM1, circTRIM1-ATG-mut and TRIM1-269aa. **C** Statistical graph of colony formation assays showing the influence of circTRIM1, circTRIM1-ATG-mut and TRIM1-269aa on TNBC chemoresistance. **D** The effects of circTRIM1, circTRIM1-ATG-mut and TRIM1-269aa on DOX-induced apoptosis were verified by flow cytometry. **E** Effects of circTRIM1, circTRIM1-ATG-mut and TRIM1-269aa on the expression of apoptosis-related proteins after DOX treatment. Transwell (**F**) and wound healing (**G**) assays were performed to evaluate the migration abilities of TNBC cells overexpressing circTRIM1, circTRIM1-ATG-mut and TRIM1-269aa. Scale bars = 200 μm. **H**. Effects of circTRIM1, circTRIM1-ATG-mut and TRIM1-269aa on EMT-related proteins. *P < 0.05; **P < 0.01; ***P < 0.001

As we demonstrated that circTRIM1 knockdown could inhibit the chemoresistance, migration and invasion abilities of TNBC cells, we further explored whether circTRIM1 or TRIM1-269aa is responsible for these biological functions. The circTRIM1 OV and circTRIM1-ATG-mut vectors were transfected into TNBC cells to investigate the influence of translation on circTRIM1 functions, and the TRIM1-269aa-Flag linear expression vector was used to determine the functions of TRIM1-269aa directly. The mRNA expression efficiencies of the vectors and of circTRIM1, TRIM1-269aa and TRIM1 were evaluated by qRT–PCR (Figure S3.A, B). We also explored the effects of the vectors on the chemoresistance of TNBC cells by MTT (3-(4,5-dimethylthiazol-2-yl)-2,5-diphenyltetrazolium bromide) and colony formation assays, which showed that circTRIM1 overexpression enhanced DOX resistance, while the mutation of ATG eliminated this effect, indicating that the translation of circTRIM1 was crucial for its oncogenic role. Moreover, direct overexpression of TRIM1-269aa via a linear expression vector had a similar effect on circTRIM1, suggesting that TRIM1-269aa is a functional translation product of circTRIM1 in TNBC chemoresistance (Fig. 4A–C, Figure S3.C, D). Flow cytometry and western blot assays also demonstrated that TRIM1-269aa, but not circTRIM1, could inhibit DOX-induced apoptosis of TNBC cells (Fig. 4D and E). In addition, Transwell assays (Fig. 4F, Figure S3.E) and wound healing assays (Fig. 4G, Figure S3.F) demonstrated that the translational activity of circTRIM1 plays a pivotal role in regulating the migration and invasion of TNBC cells. Furthermore, the expression levels of EMT-related proteins were detected, which indicated that circTRIM1 could activate the EMT pathway by encoding TRIM1-269aa (Fig. 4H). In conclusion, our results demonstrated that TRIM1-269aa, but not circTRIM1, promotes the chemoresistance, migration and invasion of TNBC cells.

### TRIM1-269aa can be transmitted by exosomes to exhibit oncogenic functions in TNBC cells

**Fig. 5:**
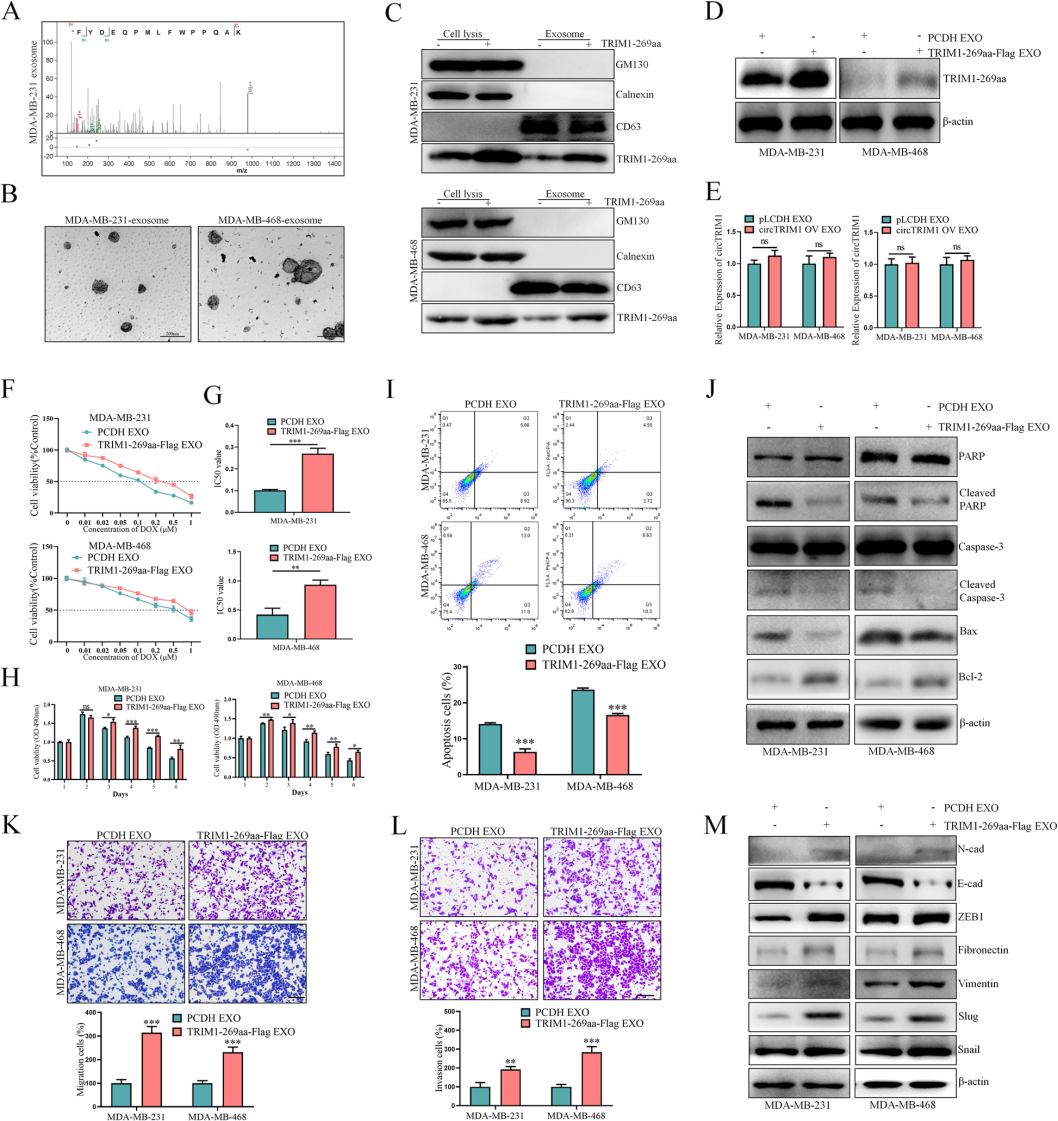
TRIM1-269aa can be transmitted by exosomes to exert its oncogenic effects.** A** The LC‒MS data of MDA-MB-231 exosomes were downloaded from the iProX database, and the specific peptide sequence of TRIM1-269aa was identified. **B** Exosomes isolated from TNBC cells were imaged by electron microscopy. **C** The exosome markers were examined by western blotting. **D** TRIM1-269aa expression in pLCDH-exosome- or TRIM1-269aa-exosome-treated TNBC cells. **E** Left panel: The exosomes of TNBC cells with or without circTRIM1 overexpression were extracted, and the relative expression of circTRIM1 in exosomes were detected by qRT-PCR. Right panel: The exosomes of TNBC cells with or without circTRIM1 overexpression were extracted to further treat MDA-MB-231 and MDA-MB-468 cells, and the effects of exosomes on circTRIM1 expression were examined. **F** Dose-dependent effects of TRIM1-269aa-exosome on TNBC chemoresistance. **G** Effect of the TRIM1-269aa-exosome on the IC50 of TNBC cells. **H** Time-dependent effects of the TRIM1-269aa-exosome on TNBC chemoresistance. Flow cytometry (**I**) and western blot (**J**) assays verified the effects of the TRIM1-269aa-exosome on DOX-induced TNBC cell apoptosis. The migration (**K**) and invasion (**L**) abilities of TNBC cells after pLCDH exosome or TRIM1-269aa-exosome treatment. Scale bars = 200 μm. **M**. EMT-related proteins were examined by western blotting. ns, nonsignificant; *P < 0.05; **P < 0.01; ***P < 0.001

Previous studies have shown that some DNAs, RNAs and proteins can be packaged into exosomes to perform biological functions via transmission between cells [30]. To test whether TRIM1-269aa is a component of TNBC exosomes, we first downloaded LC‒MS data for MDA-MB-231 exosomes from the iProX database (IPX0001929001) [31], and a TRIM1-269aa-specific peptide sequence (KAQPPWFLMPQEDYF) was identified, which suggested that TRIM1-269aa might be a component of TNBC exosomes (Fig. 5A). To further verify that TRIM1-269aa can be transmitted by exosomes from TNBC cells, we isolated exosomes from both MDA-MB-231 and MDA-MB-468 cells by ultracentrifugation, and the morphologies of the exosomes were photographed by transmission electron microscopy (Fig. 5B). Moreover, TRIM1-269aa was overexpressed in TNBC cells, and the expression levels of well-known exosome markers together with TRIM1-269aa in TNBC cell lysates and exosomes were detected, which confirmed the presence of TRIM1-269aa in TNBC exosomes (Fig. 5C). Furthermore, exosomes derived from TRIM1-269aa-overexpressing cells increased the expression of TRIM1-269aa in TNBC cells (Fig. 5D). We also detected whether circTRIM1 could also be transmitted by exosomes, and our results demonstrated that TRIM1-269aa, but not circTRIM1, could be packaged into exosomes and transmitted between TNBC cells (Fig. 5E).

To confirm the effects of the TRIM1-269aa-exosome on the chemoresistance of TNBC cells, cytotoxicity assays were first performed, which demonstrated that TNBC cells treated with the TRIM1-269aa-exosome exhibited increased chemoresistance to DOX treatment in a dose- and time-dependent manner (Fig. 5F-H). Flow cytometry and western blot assays further verified these results (Fig. 5I and J). Transwell and western blot assays were also performed, which demonstrated that the TRIM1-269aa-exosome promoted the migration and invasion abilities of TNBC cells (Fig. 5K-M). The above results confirmed that TRIM1-269aa could be transmitted by exosomes to exhibit oncogenic functions in TNBC cells.

### TRIM1-269aa promotes the translocation of MARCKS to activate the PI3K/AKT/mTOR pathway

**Fig. 6:**
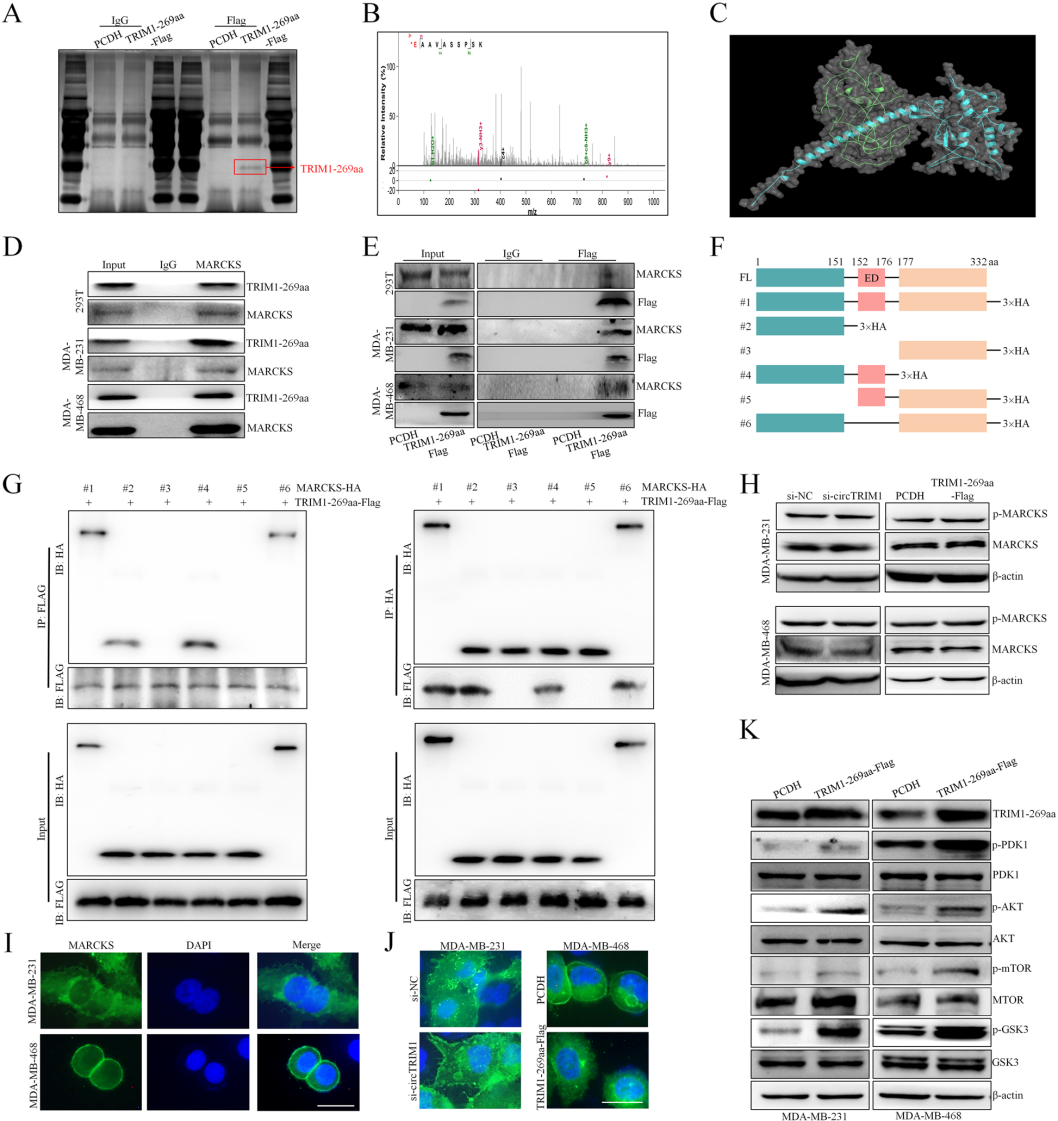
TRIM1-269aa directly interacted with and translocated MARCKS to activate the PI3K/AKT/mTOR pathway.** A** Immunoprecipitation was performed in 293T cells with TRIM1-269aa overexpression and control vectors by using IgG or an anti-FLAG antibody. **B** Specific peptide sequence of MARCKS identified by LC‒MS. **C** The 3D structures of the TRIM1-269aa and MARCKS proteins were predicted by AlphaFold, and the TRIM1-269aa/MARCKS docking was analyzed by GRAMM-X. TRIM1-269aa-Flag was transfected into 293T and TNBC cells, and the interaction between TRIM1-269aa/MARCKS was verified by co-IP with MARCKS (**D**) and Flag (**E**) antibodies. **F**. The MARCKS protein was divided into 6 truncations based on its domains with a 3×HA tag added to the C-terµinus. **G**. MARCKS truncation and TRIM1-269aa-Flag constructs were cotransfected into 293T cells. Anti-Flag antibody (left panel) and anti-HA antibody (right panel) were used to determine the interactions. **H**. TRIM1-269aa was knocked down or overexpressed in TNBC cells, and the expression levels of total MARCKS and PSD-phosphorylated MARCKS (p-MARCKS) were detected by western blot. **I**. Subcellular location of MARCKS in MDA-MB-231 and MDA-MB-468 cells. Scale bars = 20 μm. **J**. MDA-MB-231 cells were transfected with circTRIM1 siRNA, MDA-MB-468 cells were overexpressed with TRIM1-269aa-Flag, and the subcellular location of MARCKS was detected. Scale bars = 20 μm. **K**. TNBC cells were transfected with the TRIM1-269aa overexpression vector, and the activation of the PI3K/AKT/mTOR pathway was evaluated

To elucidate the molecular mechanisms of TRIM1-269aa, we initially conducted an IP assay to identify potential functional proteins that interact with TRIM1-269aa (Fig. 6A). The IP products pulled down by the Flag antibody in both the PCDH and TRIM1-269aa-Flag groups were further subjected to LC‒MS analysis. Notably, we observed that MARCKS (myristoylated alanine-rich c-kinase substrate) exhibited the highest rating among proteins that were exclusively identified in the TRIM1-269aa-Flag group (Figure S4.A). Consequently, the MARCKS protein was considered a potential downstream target of TRIM1-269aa, and the specific peptide sequence of MARCKS identified by LC‒MS is shown in Fig. 6B. The calculated 3D structures of TRIM1-269aa and MARCKS were obtained from AlphaFold [32], and the TRIM1-269aa/MARCKS docking data were analyzed by GRAMM-X software (Fig. 6C) [33]. Further IP assays were repeated in 293T and TNBC cells, and the TRIM1-269aa/MARCKS interaction was verified by both Flag and MARCKS antibodies (Fig. 6D and E). According to previous publications, the MARCKS protein contains three highly conserved domains, the N-terminal myristoylated domain (NMD), the multiple homology 2 domain (MH2), and the phosphorylation site domain (PSD); the PSD region is the most crucial effector domain for the functions of MARCKS, which can be regulated by phosphorylation or CaM bunding [34]. Based on the structure of MARCKS, we divided the MARCKS protein into N-terminal, PSD and C-terminal regions, and full-length or truncated MARCKS vectors with 3×HA tags were constructed (Fig. 6F). Similar to previous reports, the calculated molecular weight (MW) of human MARCKS was 31.56 kDa, but its observed MW was approximately 80 kDa in TNBC cells [35], and MARCKS truncation product 6, with a calculated MW of 28.50 kDa, showed a similar observed MV. For truncation products 2–5, the observed MWs were similar to the calculated MWs in TNBC cells, which were 14.09, 14.42, 17.15 and 14.49 kDa, respectively. The vectors were transfected into 293T cells, and co-IP assays were performed to detect the interaction between TRIM1-269aa and full-length or truncated MARCKS proteins. As shown in Fig. 6G, our results showed that TRIM1-269aa only interacted with MARCKS truncations 1, 2, 4 and 6, indicating that the N-terminus of MARCKS, which has been reported to account for the binding of other proteins, was crucial for its interaction with TRIM1-269aa [36].

Based on previous publications, the functions of MARCKS are closely correlated with its subcellular location in cells, which could be controlled by either PKC/RhoA-dependent phosphorylation or CaM binding. Because we confirmed the binding of TRIM1-269aa with MARCKS, we assessed the influence of TRIM1-269aa on the MARCKS protein. The expression and phosphorylation of the MARCKS protein were first detected, and we found that knockdown of circTRIM1 or overexpression of TRIM1-269aa did not influence the total protein expression or the phosphorylation of MARCKS at the PSD (Fig. 6H). We further detected the subcellular location of MARCKS in TNBC cells and found that MARCKS was predominantly located in the cytoplasm of MDA-MB-231 cells, whereas it was primarily found on the plasma membrane of MDA-MB-468 cells (Fig. 6I). To test the effects of circTRIM1 or TRIM1-269aa on the subcellular location of MARCKS, we transfected MDA-MB-231 cells with circTRIM1 siRNA and MDA-MB-468 cells with a TRIM1-269aa overexpression vector. We found that circTRIM1 knockdown promoted the translocation of MARCKS from the cytoplasm to the cytomembrane, and TRIM1-269aa overexpression had the opposite effect (Fig. 6J), confirming that circTRIM1/TRIM1-269aa influenced MARCKS translocation without regulating its phosphorylation. The subcellular location of MARCKS is correlated with its PSD phosphorylation level, and the translocation of MARCKS from the cytomembrane to the plasma could release PIP2 to activate the PI3K/AKT/mTOR axis [37]. As shown in Fig. 6K, our results demonstrated that TRIM1-269aa promoted the activation of the downstream PI3K/PI3K/AKT/mTOR pathway. These results demonstrated that TRIM1-269aa promoted the translocation of MARCKS in a non-phosphorylation-dependent manner, which further activated the PI3K/PI3K/AKT/mTOR pathway.

### TRIM1-269aa promotes the malignant behaviors of TNBC cells by enhancing calmodulin-induced MARCKS translocation

**Fig. 7:**
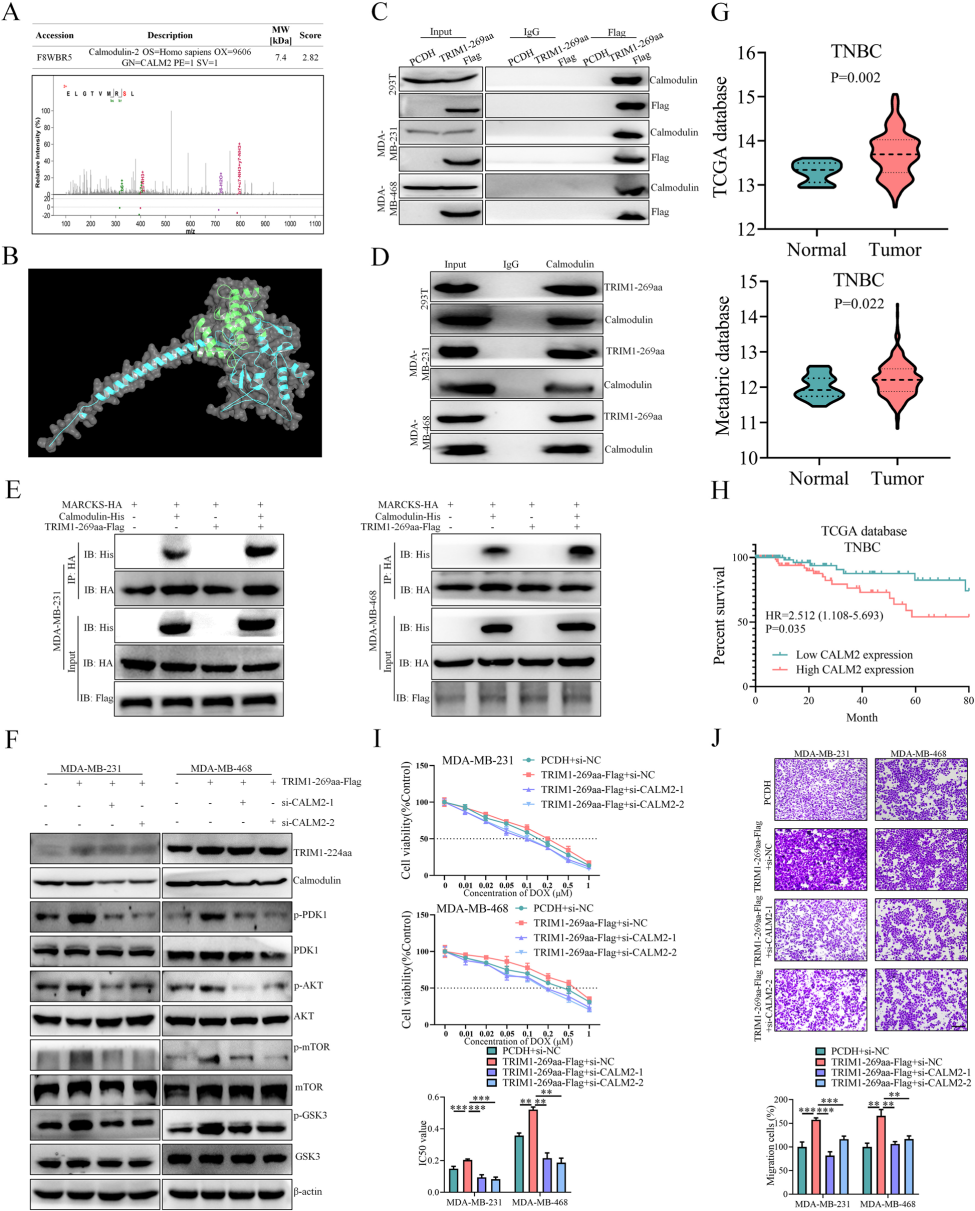
TRIM1-269aa promotes the malignant behaviors of TNBC cells by enhancing calmodulin-induced MARCKS translocation.** A** Specific peptide sequence of calmodulin identified in the LC‒MS data shown in Fig. 6A. **B** The 3D structures of the TRIM1-269aa and calmodulin proteins were predicted by AlphaFold, and the TRIM1-269aa/calmodulin docking data were analyzed by GRAMM-X software. **C** TRIM1-269aa-Flag was transfected into 293T and TNBC cells, and the interaction between TRIM1-269aa and calmodulin was verified using an anti-Flag antibody. **D** The interaction between TRIM1-269aa and calmodulin was examined in 293T and TNBC cells by using an anti-calmodulin antibody. **E** Co-IP and subsequent western blotting assays verified that TRIM1-269 restored the binding of MARCKS to calmodulin. **F** TNBC cells were transfected with a TRIM1-269aa overexpression vector or CALM2 siRNAs, and the activation of the PI3K/AKT/mTOR pathway was verified. **G** TCGA and Metabric databases confirmed that CALM2 was overexpressed in TNBC tumor tissues. **H** TCGA database analysis showed that high CALM2 expression was an unfavorable prognostic factor in patients with TNBC. The effects of TRIM1-269aa and CALM2 siRNAs on TNBC chemoresistance (**I**) and migration (**J**) were further examined. Scale bars = 200 μm. **P < 0.01; ***P < 0.001

 As previously reported, MARCKS translocation was correlated with either PKC/RhoA-dependent PSD phosphorylation or calcium-dependent calmodulin binding [38], and we further analyzed the results shown in Fig. 6A to filter potential proteins related to MARCKS translocation. As shown in Fig. 7A, upper panel, we discovered that calmodulin, which can be encoded by the CALM1/2/3 genes, was enriched with the TRIM1-269aa antibody, which might account for the TRIM1-269aa-induced MARCKS translocation in TNBC cells. The sequences of the calmodulin peptides identified by LC‒MS are shown in Fig. 7A, lower panel. The TRIM1-269aa/calmodulin docking site was then predicted by AlphaFold and GRAMM-X software (Fig. 7B) [32, 33]. Further co-IP assays in 293T and TNBC cells confirmed the interaction between TRIM1-269aa and calmodulin (Fig. 7C and D). Because our above results demonstrated that TRIM1-269aa could bind to both MARCKS and calmodulin, we further evaluated whether TRIM1-269aa could facilitate the interaction between MARCKS and calmodulin. As shown in Fig. 7E, we found that MARCKS could bind calmodulin and that its binding ability could be enhanced by TRIM1-269aa overexpression. As calmodulin binding to MARCKS is essential for its translocation [34], we hypothesized that calmodulin might participate in TRIM1-269aa-induced MARCKS translocation and downstream PI3K/AKT/mTOR pathway activation. TNBC cells were transfected with the TRIM1-269aa overexpression vector and/or CALM2 siRNA, and the subcellular location of MARCKS was detected, which demonstrated that CALM2 expression was crucial for MARCKS translocation from the membrane to the plasma of TNBC cells (Figure S4.B). Moreover, calmodulin expression and PI3K/AKT/mTOR pathway activation were detected, which indicated that calmodulin participates in the molecular mechanism of TRIM1-269aa (Fig. 7F).

Furthermore, CALM2 was overexpressed in tumor tissues and correlated with poorer prognosis in patients with breast cancer (Figure S4.C, D) and TNBC (Fig. 7G and H) patients in the TCGA [39] and Metabric [40] databases, suggesting that CALM2 gene expression might also account for the malignant behaviors of TRIM1-269aa. We thus evaluated the influence of CALM2 knockdown on the malignant behaviors of TRIM1-269aa-overexpressing TNBC cells, and our results showed that CALM2 knockdown could eliminate the oncogenic effects of TRIM1-269aa overexpression on TNBC chemoresistance and metastasis (Fig. 7I and J, Figure S4.E–I). Taken together, our results demonstrated that TRIM1-269aa could enhance the interaction between MARCKS and calmodulin to facilitate the translocation of MARCKS, further promoting PI3K/AKT/mTOR pathway activation and malignant behaviors in TNBC cells.

### Specific inhibition of PI3K/AKT/mTOR activation suppresses the oncogenic functions of TRIM1-269aa

**Fig. 8:**
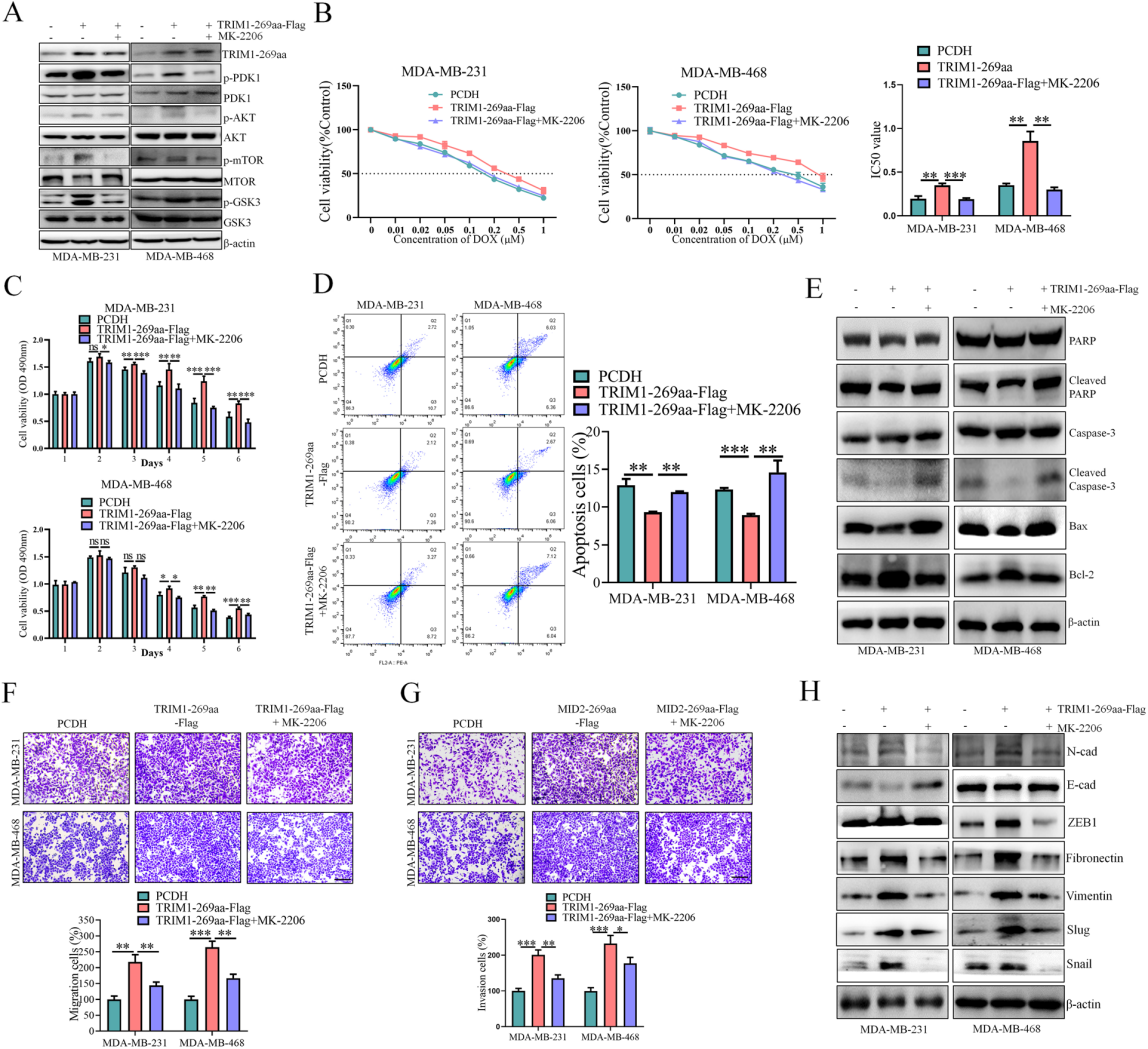
The specific inhibition of the PI3K/AKT/mTOR pathway by MK-2206 blocked the oncogenic functions of TRIM1-269aa.** A** Effects of MK-2206 on TRIM1-269aa-induced activation of the PI3K/AKT/mTOR pathway. **B** Inhibition of the PI3K/AKT/mTOR pathway suppressed the chemoresistance of TRIM1-269aa-overexpressing TNBC cells in a dose-dependent manner. **C** MK-2206 suppressed the chemoresistance of TRIM1-269aa-overexpressing TNBC cells in a time-dependent manner. **D** MK-2206 treatment facilitates DOX-induced apoptosis in TRIM1-269aa-overexpressing TNBC cells. **E** TRIM1-269aa-overexpressing TNBC cells were treated with MK-2206, and the expression levels of apoptosis-related proteins were detected. Effects of TRIM1-269aa and MK-2206 on the migration (**F**) and invasion (**G**) abilities of TNBC cells. Scale bars = 200 μm. **H**. EMT-related proteins were examined in TRIM1-269aa-overexpressing TNBC cells after treatment with MK-2206. *P < 0.05; **P < 0.01; ***P < 0.001

 Our results revealed that TRIM1-269aa could promote CaM-dependent MARCKS translocation to activate the PI3K/AKT/mTOR pathway, which is important for malignant cancer behaviors, including chemoresistance and metastasis [41]; thus, we further explored whether inhibiting PI3K/AKT/mTOR activation could eliminate the oncogenic functions of TRIM1-269aa. As shown in Fig. 8A, we verified that MK-2206, a specific inhibitor of AKT, could effectively suppress TRIM1-269aa-induced PI3K/AKT/mTOR activation. In vitro experiments were further performed to evaluate the effects of MK-2206 on the oncogenic effects of TRIM1-269aa, which demonstrated that MK-2206 could inhibit the TRIM1-269aa-induced chemoresistance and metastasis of TNBC cells (Fig. 8B–H). In conclusion, our results demonstrated that inhibition of PI3K/AKT/mTOR activation could suppress the effects of TRIM1-269aa on TNBC chemoresistance and metastasis.

### CircTRIM1 and TRIM1-269aa promote the chemoresistance and metastasis of TNBC cells in vivo

**Fig. 9:**
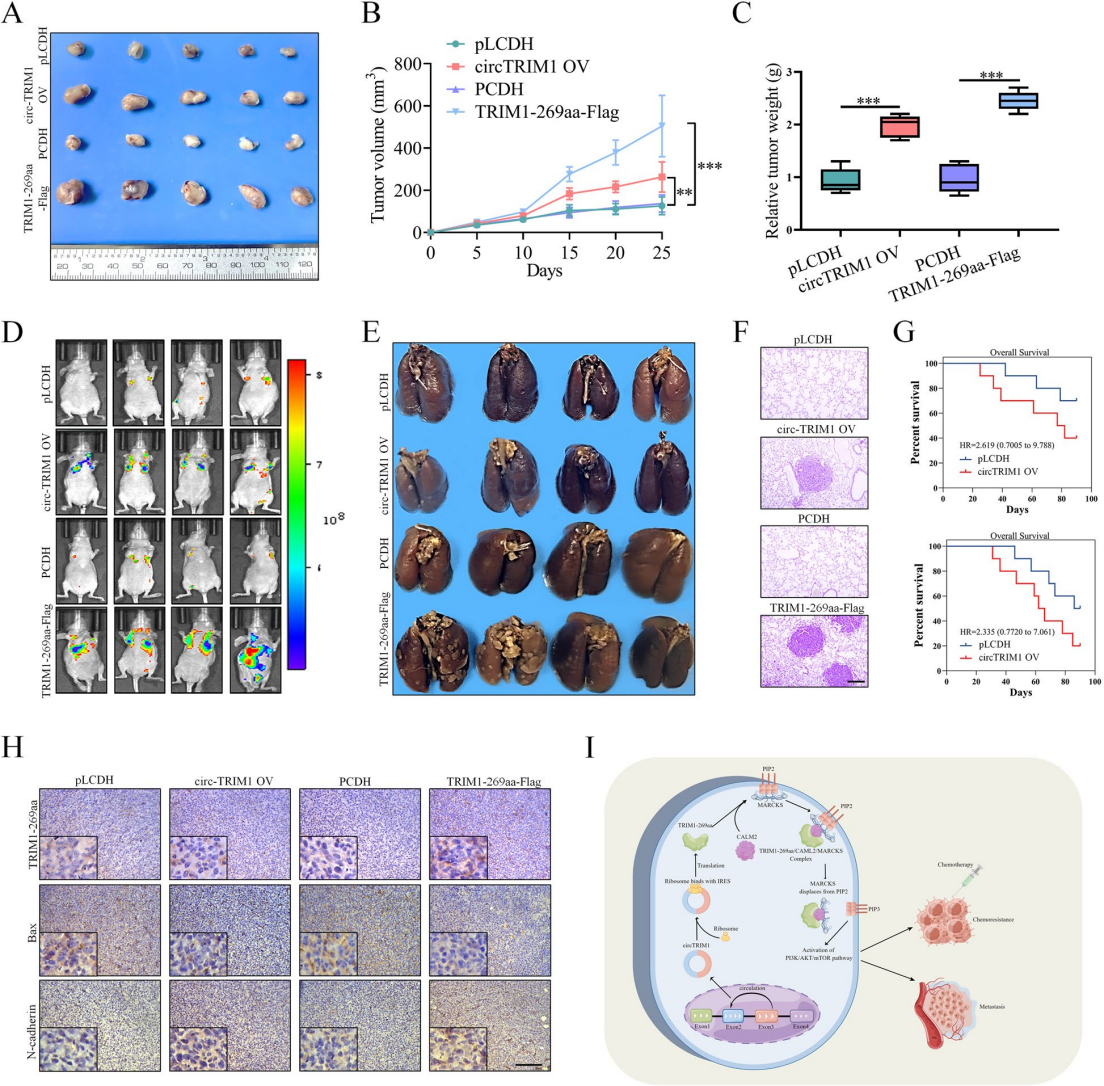
circTRIM1 and TRIM1-269aa promoted the chemoresistance and metastasis of TNBC cells in vivo.** A** MDA-MB-231 cells overexpressing circTRIM1 or TRIM1-269aa were implanted into the flanks of BALB/c mice, the mice were then injected with DOX (1 mg•kg-1) on Days 5, 10, 15, 20 and 25, and xenograft tumors were obtained from the BALB/c nude mice at the experimental endpoint. **B** Growth curves of the tumor volumes, which were measured every 5 days. **(C)** Weights of xenograft tumors at the experimental endpoint. **D** Animal in vivo imaging showing lung metastasis. **E** Images of lung metastatic nodules from BALB/c nude mice at the experimental endpoint. **F** H&E staining showing tumor metastasis. Scale bars = 200 μm. **G** Survival of mice injected with circTRIM1- or TRIM1-269-overexpressing MDA-MB-231 cells. **H** TRIM1-269aa, Bax and N-cadherin staining by IHC in xenograft tumors. Scale bars = 100 μm.  Schematic diagram showing the mechanism by which circTRIM1 encodes TRIM1-269aa in TNBC. **P < 0.01; ***P < 0.001

 To investigate the roles of circTRIM1 and TRIM1-269aa in vivo, we implanted MDA-MB-231 cells stably overexpressing circTRIM1 or TRIM1-269aa and control cells into the flanks of BALB/c mice, and the mice were injected with DOX (1 mg•kg-1) on Days 5, 10, 15, 20 and 25. As shown in Fig. 9A–C, both the circTRIM1 and TRIM1-269aa overexpression groups exhibited increased tumor volume and weight, indicating that circTRIM1 and TRIM1-269aa could promote the chemoresistance of TNBC cells in vivo. We also evaluated the effects of circTRIM1 and TRIM1-269aa on TNBC metastasis via intravenous injection of MDA-MB-231 cells. In vivo fluorescence and lung tissue imaging indicated that circTRIM1 and TRIM1-269aa overexpression aggravated the lung colonization of TNBC cells in nude mice (Fig. 9D and E, Figure S4.J). Hematoxylin–eosin staining of the lungs revealed large numbers of tumor nodes in the circTRIM1 and TRIM1-269aa overexpression groups (Fig. 9F). Furthermore, the overexpression of circTRIM1 and TRIM1-269aa significantly decreased the survival time of the mice (Fig. 9G). IHC assays were performed to detect the expression of TRIM1-269aa, Bax and N-cadherin in the tumors shown in Fig. 9A. As shown in Fig. 7H, the expression levels of Bax were decreased in circTRIM1- and TRIM1-269aa-overexpressing TNBC cells, while N-cadherin expression was increased. Taken together, our results demonstrated that circTRIM1 and TRIM1-269aa could promote the chemoresistance of TNBC cells to metastasis in vivo.

## Discussion

Over the past few decades, circRNAs have gradually become the focus of research on various diseases due to their unique properties. Advancements in high-throughput RNA sequencing and circRNA-specific molecular tools have revealed that numerous circRNAs are abnormally expressed in a wide spectrum of cancer types, suggesting potential functional roles of these circRNAs in cancers [42]. The noncoding functions of circRNAs, including their functions as miRNA-binding sponges and interactions with RBPs, have been the most extensively studied in the field of circRNA investigation [43]. For example, circ-TRIO could combine with miR-432-5p to promote tumor progression of TNBC, circROBO1 and circEZH2 functioned as protein scaffold to facilitate liver metastasis of breast cancer [44–46]. With continuous in-depth research on circRNAs, recent studies have focused on the translation abilities of circRNAs instead of their noncoding functions, as researchers have suggested that the coding potential of these circRNAs is largely underestimated [47]. Bozzoni et al. first published a novel protein encoded by circ-ZNF609 in myoblasts [48], and Kadener et al. reported direct evidence of a circMbl-encoded protein [49]. In breast cancer, a few studies have shown that circRNAs encode peptides, including circ-EIF6, which encodes EIF6-224aa [16]; circ-HER2, which encodes HER2-103 [50]; and circSEMA4B, which encodes SEMA4B-211aa [51], which reportedly participate in the progression and drug resistance of tumors. However, although the coding roles of circRNAs have remained largely underestimated, only a few studies have investigated this topic; thus, circRNA-encoded proteins are a vast untapped treasure trove in tumor research.

TNBC is a subtype characterized by the absence of estrogen receptor, progesterone receptor, and human epidermal growth factor receptor 2 expression and is considered one of the most lethal subtypes of breast cancer [52]. TNBC frequently develops resistance despite the initial response to chemotherapy, leading to treatment failure and poor prognosis [53]; therefore, understanding the mechanisms underlying chemotherapy resistance in TNBC is crucial for developing effective therapeutic strategies. DOX (doxorubicin) is the most frequently used chemical drug in the clinical treatment of TNBC; thus, we selected MDA-MB-231 cells and the DOX-resistant subcell line 231/DOX to filter potential functional coding circRNAs. Because the translation process of circRNAs requires the involvement of ribosomes, we performed translatome sequencing to identify potential functional ribosome-binding circRNAs after total RNA-seq between the MDA-MB-231 and 231/DOX cell lines in the present study. We identified a novel circRNA, circTRIM1, which was significantly highly expressed in the total and ribosome-binding RNA of 231/DOX cells. Moreover, circTRIM1 was overexpressed in chemoresistant TNBC tissues and correlated with the clinicopathological characteristics and poor prognosis of patients with TNBC. The up-regulation of circTRIM1 in chemoresistant TNBC cells and tissues might due to the elevated TRIM1 gene transcription or increased cicRNA biogenesis via circularization, and further experiments were needed to clarify the mechanisms. In breast cancer, TRIM1 expression has been reported to be up-regulated via MORC4/STAT3-mediated transcription activation, which might be the reason that leaded to circTRIM1 overexpression in chemoresistant TNBC cells and tissues [54]. By further evaluating the coding ability of circTRIM1, we confirmed that circTRIM1 could be translated into a 269-aa peptide termed TRIM1-269aa in a cap-independent manner by using a cis-regulatory element termed the IRES. Internal ribosome entry sites (IRESs) can recruit ribosomes to RNA sequences, which is the most commonly reported mechanism of circRNA translation, and IRES-driven translation of circRNAs has been reported in multiple cancers [55]. Another important cap-independent translation mechanism of circRNA involves m6A modification. Short sequences with m6A-induced ribosome engagement sites (MIRESs) can function as IRES-like elements to drive circRNA translation, and a single m6A modification is sufficient to initiate circRNA translation with the initiation factor eIF4G2 and the m6A reader protein YTHDF3 [25]. In this study, we also knocked down YTHDF3 expression to verify its effects on circTRIM1 translation, and our results indicated that circTRIM1 translation could not be efficiently regulated by m6A modification. Furthermore, detection of TRIM1-269aa using a specific antibody confirmed the endogenous expression of TRIM1-269aa in TNBC cell lines and tissues, and TRIM1-269aa was overexpressed in chemoresistant TNBC tissues, consistent with circTRIM1. The above results indicate that circTRIM1 and its translation product TRIM1-269aa might be oncogenic factors in TNBC.

In vitro and in vivo studies were further performed and demonstrated that circTRIM1 plays central roles in regulating the chemoresistance and metastasis of TNBC cells by encoding TRIM1-269aa, which can be packaged into exosomes to transmit its oncogenic abilities between cells. Moreover, RNA-seq and enrichment analyses revealed that the biological functions of circTRIM1 and TRIM1-269aa were closely correlated with the progression of TNBC cell apoptosis, which might account for the chemoresistance of TNBC cells. Taken together, our results demonstrated that circTRIM1 encodes TRIM1-269aa to promote chemoresistance and metastasis in TNBC cells.

To explore the molecular mechanism by which circTRIM1 encodes TRIM1-269aa, co-IP and MS were performed, and the results confirmed that MARCKS is a direct downstream target of TRIM1-269aa. The myristoylated alanine-rich c kinase substrate (MARCKS) protein is a highly conserved intracellular protein that plays crucial roles in various cellular processes, such as exocytosis, cell migration, and regulation of the inflammatory response [56]. The rod-shaped MARKCS protein contains three distinct evolutionarily conserved regions: the N-terminal myristoylated domain (NMD), the multiple homology 2 domain (MH2), and the phosphorylation site domain (PSD) (also known as the effector domain (ED)) [57]. In cancers, MARCKS functions as a double-edged sword. For instance, Wenzel T et al. reported that CRC cells lacking MARCKS expression exhibit enhanced chemoresistance due to increased ABCB1 expression [58]; moreover, MARCKS protein expression levels are inversely correlated with GBM proliferation and intracranial xenograft growth rates in vivo [59]. In addition, the oncogenic roles of MARCKS in cancer proliferation, metastasis and chemoresistance have also been demonstrated [38]. The roles of MARCKS are strongly correlated with its subcellular location in cells, as membrane-located MARCKS interacts with PI [4, 5]P_2_; once MARCKS is translocated into the cytoplasm, PI [4, 5]P_2_ is released from MARCKS to further interact with other proteins and activate downstream pathways, such as the PI3K/AKT/mTOR pathway [60]. It has been reported that the translocation of MARCKS from the plasma membrane to the cytoplasm could be controlled by either PKC/RhoA-dependent phosphorylation at the PSD or calmodulin binding [61]. For example, PKC-alpha and PKC-delta can phosphorylate and translocate MARCKS from the plasma membrane to the cytoplasm in smoke-related lung cancer, and the binding of MARCKS to calmodulin (CaM) could also result in the translocation of MARCKS to activate the downstream PI3K/AKT/mTOR pathway [34, 62]. We thus detected the effects of circTRIM1 and TRIM1-269aa on the phosphorylation levels, subcellular locations and PI3K/PI3K/AKT/mTOR pathway activation of MARCKS. We found no significant effect on MARCKS phosphorylation after circTRIM1/TRIM1-269aa overexpression or knockdown, but its subcellular location and activation of the PI3K/PI3K/AKT/mTOR pathway were markedly altered, suggesting that TRIM-269aa might promote chemoresistance and metastasis in TNBC cells by influencing the interaction between MARCKS and calmodulin to activate the PI3K/AKT/mTOR pathway. Furthermore, a specific AKT inhibitor (MK-2206) was used, and we demonstrated that the phosphorylation of MARCKS and activation of the PI3K/AKT/mTOR pathway were crucial for the oncogenic functions of TRIM1-269aa. In conclusion, the results demonstrated that TRIM1-269aa could directly interact with MARKCS, further activating the PI3K/AKT/mTOR pathway to promote the chemoresistance and metastasis of TNBC cells, which might correlate with the functions of calmodulin.

By further investigating the molecular mechanisms of MID-269aa-induced MARCKS translocation, we found that calmodulin was another downstream target that directly interacted with TRIM1-269aa. CaM can be encoded by a member of the CALM gene family (CALM1, CALM2, and CALM3), which is a Ca^2+^-binding protein with high affinity for the MARCKS protein [63]. Previous studies have reported that calmodulin shares the same MARCKS PSD that serves as the site for PKC phosphorylation [64], and the combination of MARCKS PSD with calmodulin leads to its dissociation from the plasma membrane and subsequent modulation of cellular processes such as cytoskeletal reorganization, membrane trafficking and activation of the PI3K/AKT/mTOR pathway [65]. The CaM-MARCKS axis is involved in multiple cellular events, including cell adhesion, migration, and exocytosis [66, 67]. Moreover, dysregulation of this pathway has been implicated in several diseases, such as cancer and neurodegenerative disorders [56, 68]. In our study, we confirmed that TRIM1-269aa could enhance the interaction between calmodulin and MARCKS, which accounted for the TRIM1-269aa-induced MARCKS translocation and activation of the PI3K/AKT/mTOR pathway. Moreover, we also demonstrated that calmodulin expression was crucial for the oncogenic effects of TRIM1-269aa. Taken together, our results proved that TRIM1-269aa could enhance the interaction between MARCKS and calmodulin to translocate MARCKS from the cytomembrane to the cytoplasm, which further promoted both the activation of the PI3K/AKT/mTOR pathway and the oncogenic behaviors of TNBC cells.

In summary, our study revealed the crucial roles of circTRIM1 in regulating TNBC chemoresistance and metastasis. CircTRIM1 encodes a novel 269-aa protein termed TRIM1-269aa, which promotes the translocation of MARCKS by enhancing the interaction between MARCKS and calmodulin. The translocated MARCKS could further displace from the cell membrane and initiate the activation of downstream PI3K/AKT/mTOR pathways (Fig. 7I). Clinically, circTRIM1 was closely associated with poor prognosis and clinicopathological characteristics in patients with TNBC. Taken together, our study demonstrated that circTRIM1 is a potential biomarker for TNBC diagnosis and prognosis. Targeting circTRIM1 and TRIM1-269aa could be effective strategies for overcoming TNBC chemoresistance and metastasis.

## Materials and methods

### Ethics statement and human tissue samples

The experimental procedures conducted in this study received approval from the Ethical Committee of Shandong University. Tissue samples were obtained from patients admitted to Qilu Hospital at the time of surgery and promptly stored at -80 °C. Written informed consent was obtained from all patients, allowing the utilization of these clinical materials for research purposes.

### Cell lines and vectors

All cell lines were obtained from the American Type Culture Collection (Manassas, VA, USA) and were maintained using standard media and conditions. All cell lines were grown at 37 C in a 5% CO_2_ cell culture incubator. Vectors were synthesized from Vigene Biosciences (Rockville, MD, USA).

### RNA-seq (transcriptome sequencing)

MDA-MB-231 and its metastatic subcell line 231_M were used for sequencing analysis. The sample preparation and circRNA sequencing were performed using Lc-Bio Technologies (Hangzhou, China). Significantly differentially expressed circRNAs were retained by screening for fold change R 2.0 and *p* < 0.05.

### RNC-seq (translatome sequencing)

RNC-seq was performed as previously reported in this study [69]. Briefly, cells were subjected to a pre-treatment step with 100 µg/ml of cycloheximide for 15 min. Subsequently, the cells underwent pre-chilled PBS washes, and 2 ml of cell lysis buffer [1% Triton X-100 in ribosome buffer (RB buffer): 20 mM HEPES-KOH (pH 7.4), 15 mM MgCl2, 200 mM KCl, 100 µg/ml cycloheximide, and 2 mM dithiothreitol] was added. To eliminate cell debris, the samples were centrifuged at 16,200 g for 10 min at 4 °C. After centrifugation, the supernatants were carefully transferred onto the surface of 20 ml of sucrose buffer (containing 30% sucrose in RB buffer). RNCs were pelleted after ultra-centrifugation at 185,000 g for 5 h at 4 °C, and significantly differentially expressed circRNAs were retained by screening for fold change R 2.0 and *p* < 0.05.

### In situ hybridization (ISH)

A specific digoxin-labeled circTRIM1 probe was designed, and the tumor samples were fixed with formalin, embedded in paraffin, and sectioned into 6-µm slides. The ISH assay was performed using the Enhanced Sensitive ISH Detection kit I (BOSTER, Wuhan, China) following the manufacturer’s protocol.

### Actinomycin D and RNase R

Transcription was prevented by the addition of 2 mg/mL actinomycin D (Sigma-Aldrich, USA) or DMSO (no treatment [Mock]) (Sigma-Aldrich, USA) as the negative control for the indicated times. RNase R was used to identify and confirm the character of the circRNA. The RNAs extracted from MDA-MB-231 or MDA-MB-468 cells were divided into two equal parts, respectively, one for RNase R and the other for Mock. Total RNA (2 mg) was incubated with 3 U/mg of RNase R for 30 min at 37 C. After treatment with actinomycin D and RNase R, the RNA expression levels of circTRIM1 and TRIM1 mRNA were detected by using qRT-PCR.

### Fluorescence in situ hybridization (FISH)

The FISH assay was performed using the FISH kit from GenePharma, Shanghai, China, to determine the subcellular localization of circTRIM1 in TNBC cells. The cells were prehybridized at 73 °C for 5 min, followed by hybridization with specific Cy3-labeled circTRIM1 probes at 37 °C overnight. To visualize the cell nuclei, Hoechst staining was employed. Photographs of the cells were captured using a fluorescence microscope (Leica, Wetzlar, Germany).

### Polysome profiling assay

Sucrose gradient fractionation assays were conducted following a previously described protocol [16]. In brief, 293T cells were transfected with circTRIM1 or circTRIM1-ATG-mut vectors for 48 h in 10 cm plates. Subsequently, the cells were treated with 100 µg/mL CHX for 15 min and washed twice with cold PBS. The cells were then harvested and lysed using 500 µL of lysis buffer containing 5 mM Tris-HCl (pH 7.5), 2.5 mM MgCl2, 1.5 mM KCl, 1× protease inhibitor cocktail (EDTA free), 0.5% Triton X-100, 2 mM DTT, 0.5% sodium deoxycholate, 100 U of RNase inhibitor, and 100 µg/mL CHX for 15 min. The polysome lysate was subsequently centrifuged at 16,000 × g for 7 min, and the supernatant was collected. To prepare the sucrose gradient, a 5–50% sucrose gradient solution was prepared in an ultracentrifuge tube and stored overnight at 4 °C to achieve a linear gradient. The collected supernatant was layered onto the top of the sucrose gradient and centrifuged at 35,000 rpm for 2 h. After centrifugation, the solution was collected from top to bottom in 150 µL aliquots per tube, and the absorbance was measured at 254 nm using a UV spectrophotometer. Additionally, RNA was further isolated using TRIzol LS Reagent, and curve graphs depicting the distribution of circTRIM1 or circ- TRIM1-ATG-mut among the fractions were plotted according to previously reported methods.

### Luciferase reporter assay

Cells were co-transfected with RLuc-Luc vectors containing full-length or truncated IRES sequence using Lipofectamine 2000 (Invitrogen). After 48 h, luciferase activities were determined by the Dual-Luciferase Reporter Assay Kit (Promega). Results are presented as ratios of luminescence from firefly to Renilla luciferase.

### Immunohistochemistry (IHC)

Primary antibodies including TRIM1-269aa (1:100), cleaved-caspase 3 (1:200), N-cadherin (1:200) and p62 (1:200) were used to incubate paraffin slices at 4 °C overnight, following by peroxidase-conjugated secondary antibody for 2 h at room temperature. Then the tissue sections were stained with diaminobenzidine, and counterstained with hematoxylin. Olympus light microscope was used to take photos for the representative areas.

### Cell viability assay

Cell viability was determined by a 3-(4, 5-dimethylthiazol-2-yl)-2, 5-diphenyltetrazolium bromide (MTT) assay. MDA-MB-231 and MDA-MB-468 cells were cultured in 96-well plates for 6 days. After incubation for the indicated time, 20 µl of MTT (5 mg/ml in PBS) was added into each well and incubated for 4 h. The supernatants were carefully aspirated, and 100 µl of dimethyl sulfoxide was added to each well. Absorbance values at 490 nm were measured on a Microplate Reader (Bio-Rad).

### Migration and invasion assays

For the migration assay, 700 µL of medium containing 20% Fetal Bovine Serum (FBS) was added to the lower well of each chamber. Then, 1 × 10^5^ cells suspended in serum-free medium were added to the upper inserts. Following the specified incubation period, the total number of cells that adhered to the lower surface of the membrane was quantified in six representative fields. The invasion assay was conducted using a similar procedure as the migration assay, with the exception that the membrane was coated with matrigel (BD Biosciences, Bedford, MA, USA) before the cells were added to the upper inserts.

### Immunoprecipitation

The cells were lysed using a cold lysis buffer containing 1% Triton X-100, 50 mM Tris (pH 7.5), 1 mM EDTA, 150 mM NaCl, and protease inhibitors. The supernatant obtained from cell lysates was incubated with specific antibodies as indicated. Subsequently, the lysates were incubated with protein A/G beads (protein A/G agarose beads). The beads were washed with cold lysis buffer to remove any nonspecific binding, and the protein loading buffer was added to the beads. Finally, the samples were subjected to western blotting analysis to assess protein expression levels.

### LC-MS analysis

The proteins were separated on SDS-PAGE gels, and the bands of interest were carefully excised from the gel. The excised bands were then subjected to trypsin digestion using sequencing-grade trypsin (Promega, Madison, WI, USA). The resulting peptides from the digestion were analyzed using a QExactive mass spectrometer (Thermo Fisher Scientific, Carlsbad, CA, USA). The fragment spectra obtained were analyzed using the National Center for Biotechnology Information nonredundant protein database with the Mascot software (Matrix Science) for protein identification.

### In vivo proliferation and metastasis assay

Briefly, circTRIM1 or TRIM1-269aa overexpressed MDA-MB-231 cells were suspended in 200 µl of PBS: Matrigel (1:3, v/v) and subcutaneously injected into the left flank of 4–6 weeks-old BALB/c nu/nu female mice. Tumor growth was monitored by measuring the tumor diameters every 5 days. The maximum (L) and minimum (W) lengths of the tumor were measured using a slide caliper, and the tumor volume was calculated using the formula ½LW^2^. Upon completion of the experiment, the tumors were collected. For the production of experimental lung metastasis, 5 × 10^5^ cells were injected into the lateral tail veins of 4–6 weeks-old BALB/c nu/nu female mice (seven mice per group). After 4 weeks, all the mice were euthanized under anesthesia. The lungs were collected and fixed in 10% formalin. Tissue sections from the embedded samples were subjected to hematoxylin and eosin (HE) staining for tissue morphology evaluation, following previously described methods. All animal experiments were conducted with the approval of the Shandong University Animal Care and Use Committee.

### RNA extraction and qRT-PCR

Total RNA was isolated from tissues or cells using Trizol reagant (Invitrogen, USA). Then cDNA was synthesized using PrimeScript reverse transcriptase (RT) reagent kit (TaKaRa, Shiga, Japan). qRT-PCR was carried out using the SYBR green PCR mix (Takara). Actin were used as the endogenous control. The primers used in this work was supplied in Table S.1.

### Protein isolation and Western blot

Cells were lysed with RIPA buffer containing PMSF (Biocolors, Shanghai, China), separated on 10% SDS-PAGE gels, and electroblotted onto a PVDF membrane (Millipore). After blocking with 5% nonfat milk, the membrane was incubated with various specific primary antibodies overnight at 4 °C. Blots were washed and incubated with horseradish peroxidase-coupled secondary antibodies (Millipore) for 2 h. The protein bands were detected using the Pro-lighting HRP agent. β-Actin served as a loading control. The antibodies used in this study are shown in Table S.2.

### Production of the TRIM1-269aa antibody

To generate an antibody specific to TRIM1-269aa, the 252- to 269-amino acid sequence of TRIM1-269aa (EVKAQPPWFLMPQEDYFH) was selected as the template for the antigen. A cysteine (C) was added to the 5’ end of the 18-amino acid sequence to generate the antigen coupled with BSA (CEVKAQPPWFLMPQEDYFH). This modification was necessary for antibody production. The specific polyclonal antibody against the antigen sequence was produced by inoculating rabbits obtained from Abcepta (San Diego, CA, USA). This antibody is designed to recognize TRIM1-269aa specifically, thanks to the unique target sequence of the antigen. For detailed information regarding the construction procedures and inspection reports of the antibodies and antigens, please refer to Supplementary file 1, 2.

### Statistical analysis

To determine the significance of differences between two experimental groups, student’s t-test was employed. For comparisons among multiple groups, one-way analysis of variance (ANOVA) was conducted. The data used in these statistical tests followed a normal distribution, and the variances among the groups were similar. A two-tailed *p*-value less than 0.05 was considered statistically significant. The data are presented as the mean ± standard deviation (SD) and are derived from a minimum of three independent experiments. Survival analysis was performed by generating Kaplan-Meier curves, and the significance of differences was determined using the log-rank and cox test.

### Supplementary Information

Supplementary Material 1: Figure S1. A The lengths and types of circRNAs detected by transcriptome sequencing (upper panel) and translatome sequencing (lower panel) of MDA-MB-231 and 231/DOX cells. B. Basic information on codysregulated circRNAs identified via transcriptome and translatome sequencing of the 231 and 231/DOX cell lines. C. The relative expression levels of circTRIM1 and TRIM1 mRNA in TNBC cells were analyzed using qRT‒PCR after normalization to random primers and oligo dT primers. D. Relative RNA levels of circTRIM1 and TRIM1 mRNA after actinomycin D treatment measured by qRT‒PCR. E. Potential IRES sequence of circTRIM1 predicted by circBank and circRNADB. F. Upper panel: Illustration of the putative open reading frame (ORF) and IRES of circTRIM1. Lower panel: Sequences of the putative ORFs are shown in blue, the internal ribosomal entrance site (IRES) sequences are shown in purple, and other sequences are shown in green. G. The amino acid sequence of TRIM1-269aa encoded by circTRIM1 and the antigen sequence used to produce the TRIM1-269aa antibody. H. The sequences of the wild-type and mutant IRESs of circTRIM1 and the corresponding IRES structures and scores were predicted by IRESfinder. I. The translation initiation abilities of the WT or Mut IRESs were detected by dual-luciferase reporter assays. ns, nonsignificant; J. The silencing efficiencies of YTHDF3 siRNAs in TNBC cells detected by qRT-PCR. K. Effects of YTHDF3 siRNAs on protein expressions of YTHDF3 and TRIM1-269aa in TNBC cells detected by western blot. **P < 0.01; ***P < 0.001.

Supplementary Material 2: Figure S2. A. The position and sequence of circTRIM1 siRNAs. B. The efficiency of circTRIM1 siRNAs was measured by qRT‒PCR. C. The efficiency of circTRIM1 siRNAs was verified by FISH. Scale bars=40 μm. D. Effects of circTRIM1 knockdown on the protein expression of TRIM1-269aa. E. Effects of circTRIM1 knockdown on the expression of MID mRNA in TNBC cells. ns, nonsignificant; **P < 0.01; ***P < 0.001.

Supplementary Material 3: Figure S3. A. Efficiency of the transfection of TNBC cells with circTRIM1 OV, circTRIM1-ATG-mut and TRIM1-269aa-Flag. B. Effects of the transfection of TNBC cells with circTRIM1 OV, circTRIM1-ATG-mut and TRIM1-269aa-Flag on the expression of TRIM1 mRNA. C. Time-dependent effects of circTRIM1, circTRIM1-ATG-mut and TRIM1-269aa on the chemoresistance of TNBC cells. D. Colony formation assays were used to evaluate the effects of circTRIM1 OV, circTRIM1-ATG-mut and TRIM1-269aa-Flag on the chemoresistance of TNBC cells. E. Effects of circTRIM1 OV, circTRIM1-ATG-mut and TRIM1-269aa-Flag on the invasion of TNBC cells. Scale bars = 200 μm. F. Effects of circTRIM1 OV, circTRIM1-ATG-mut and TRIM1-269aa-Flag on the migration abilities of TNBC cells based on a wound healing assay. *P< 0.05; **P < 0.01; ***P < 0.001.

Supplementary Material 4: Figure S4. A.Precipitates were subjected to LC‒MS with an anti-Flag antibody, and proteins identified only in the TRIM1-269aa overexpression group are presented. B. Subcellular location of MARCKS in TNBC cells. Scale bars = 20 μm. C. The TCGA and METABRIC databases were used to analyze the expression of CALM2 in normal and tumor-confirmed breast tissues.D. High CALM2 expression suggested poorer prognosis in patients with breast cancer in the TCGA cohort. E. Knockdown of CALM2 inhibited TRIM1-269aa-promoted chemoresistance in TNBC cells in a time-dependent manner. Flow cytometry (F) and western blotting (G) confirmed that knockdown of CALM2 promoted the apoptosis of TNBC cells inhibited by TRIM1-269aa. Transwell (H) and western blot (I) assays confirmed that CALM2 silencing inhibited TRIM1-269aa-promoted invasion and EMT in TNBC cells. Scale bars = 200 μm. J. Number of lung metastases in BALB/c nude mice injected with circTRIM1- or TRIM1-269aa-overexpressing TNBC cells. ns, nonsignificant; *P < 0.05; **P < 0.01; ***P < 0.001.

Supplementary Material 5.

Supplementary Material 6.

Supplementary Material 7.

Supplementary Material 8.

## Data Availability

No datasets were generated or analysed during the current study.
