# Supplementary figures and images for: CircTRIM1 encodes TRIM1-269aa to promote chemoresistance and metastasis of TNBC via enhancing CaM-dependent MARCKS translocation and PI3K/AKT/mTOR activation
Source: Mol Cancer. 2024 May 16;23:102. doi: 10.1186/s12943-024-02019-6 (PMC11097450; doi:10.1186/s12943-024-02019-6)

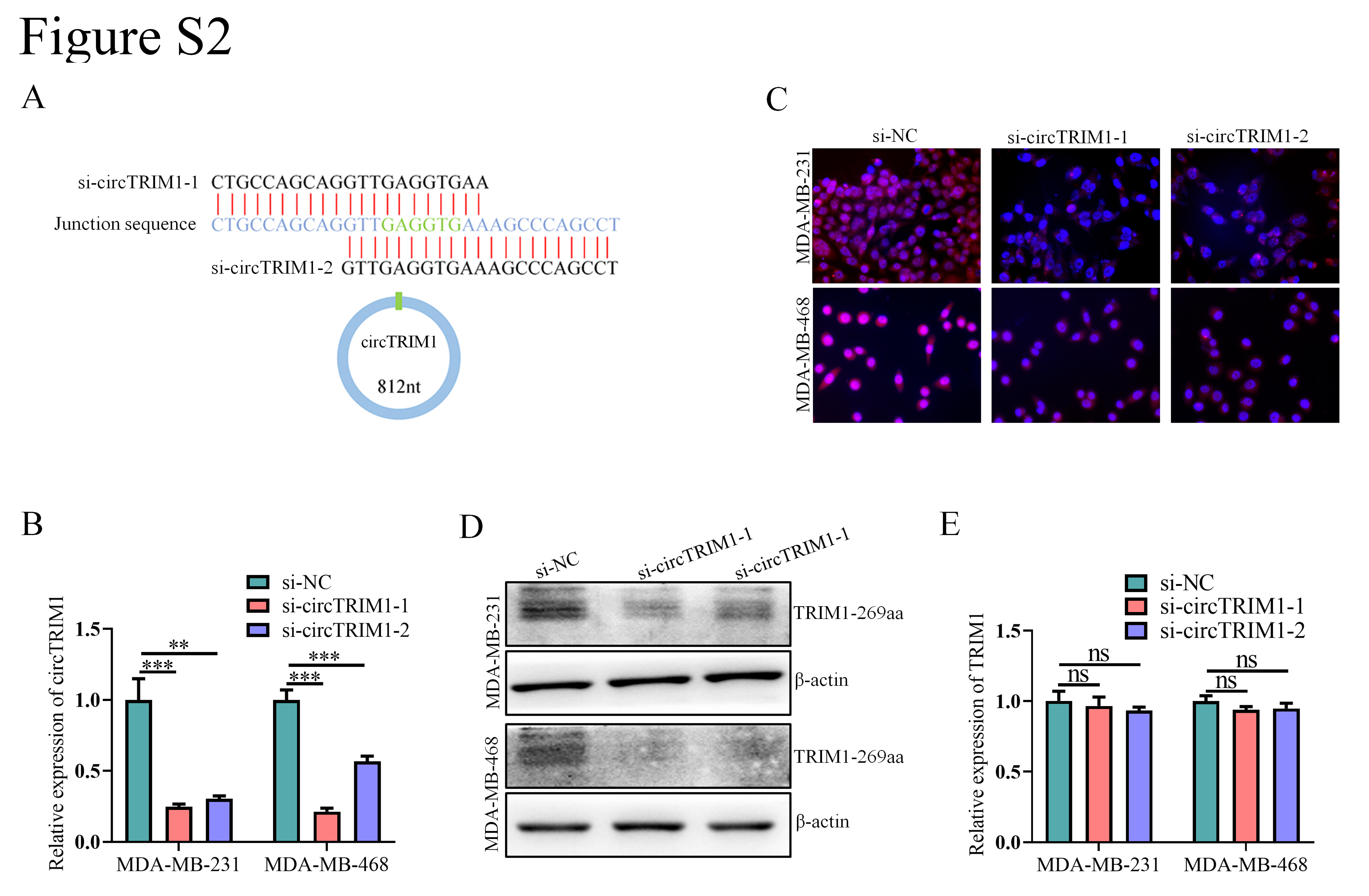

Supplement: Supplementary file 1 — Supplementary Material 1: Figure S1. A The lengths and types of circRNAs detected by transcriptome sequencing (upper panel) and translatome sequencing (lower panel) of MDA-MB-231 and 231/DOX cells. B. Basic information on codysregulated circRNAs identified via transcriptome and translatome sequencing of the 231 and 231/DOX cell lines. C. The relative expression levels of circTRIM1 and TRIM1 mRNA in TNBC cells were analyzed using qRT‒PCR after normalization to random primers and oligo dT primers. D. Relative RNA levels of circTRIM1 and TRIM1 mRNA after actinomycin D treatment measured by qRT‒PCR. E. Potential IRES sequence of circTRIM1 predicted by circBank and circRNADB. F. Upper panel: Illustration of the putative open reading frame (ORF) and IRES of circTRIM1. Lower panel: Sequences of the putative ORFs are shown in blue, the internal ribosomal entrance site (IRES) sequences are shown in purple, and other sequences are shown in green. G. The amino acid sequence of TRIM1-269aa encoded by circTRIM1 and the antigen sequence used to produce the TRIM1-269aa antibody. H. The sequences of the wild-type and mutant IRESs of circTRIM1 and the corresponding IRES structures and scores were predicted by IRESfinder. I. The translation initiation abilities of the WT or Mut IRESs were detected by dual-luciferase reporter assays. ns, nonsignificant; J. The silencing efficiencies of YTHDF3 siRNAs in TNBC cells detected by qRT-PCR. K. Effects of YTHDF3 siRNAs on protein expressions of YTHDF3 and TRIM1-269aa in TNBC cells detected by western blot. **P < 0.01; ***P < 0.001. [file 12943_2024_2019_MOESM1_ESM.jpg]

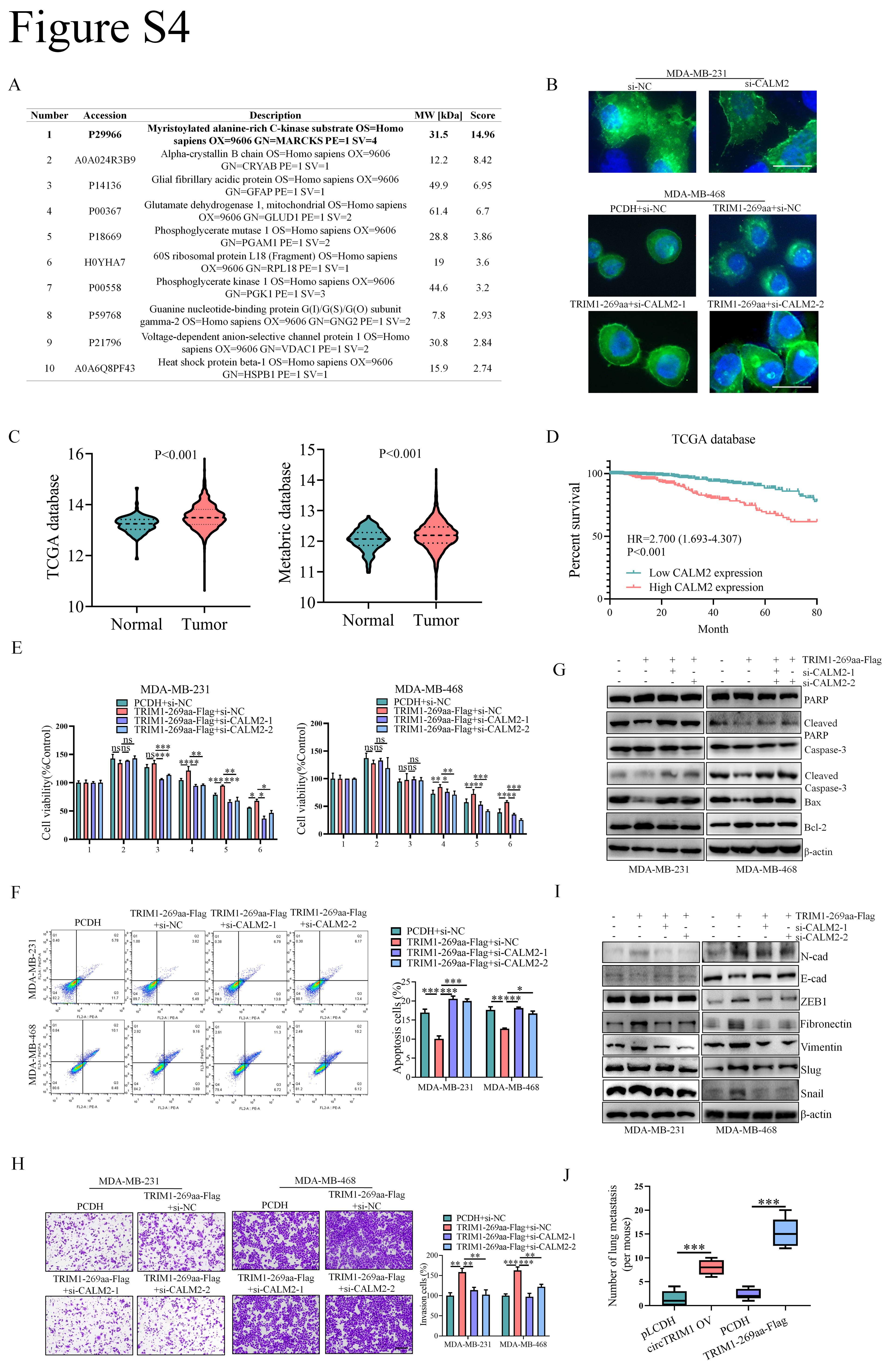

Supplement: Supplementary file 4 — Supplementary Material 4: Figure S4. A.Precipitates were subjected to LC‒MS with an anti-Flag antibody, and proteins identified only in the TRIM1-269aa overexpression group are presented. B. Subcellular location of MARCKS in TNBC cells. Scale bars = 20 μm. C. The TCGA and METABRIC databases were used to analyze the expression of CALM2 in normal and tumor-confirmed breast tissues.D. High CALM2 expression suggested poorer prognosis in patients with breast cancer in the TCGA cohort. E. Knockdown of CALM2 inhibited TRIM1-269aa-promoted chemoresistance in TNBC cells in a time-dependent manner. Flow cytometry (F) and western blotting (G) confirmed that knockdown of CALM2 promoted the apoptosis of TNBC cells inhibited by TRIM1-269aa. Transwell (H) and western blot (I) assays confirmed that CALM2 silencing inhibited TRIM1-269aa-promoted invasion and EMT in TNBC cells. Scale bars = 200 μm. J. Number of lung metastases in BALB/c nude mice injected with circTRIM1- or TRIM1-269aa-overexpressing TNBC cells. ns, nonsignificant; *P < 0.05; **P < 0.01; ***P < 0.001. [file 12943_2024_2019_MOESM4_ESM.jpg]

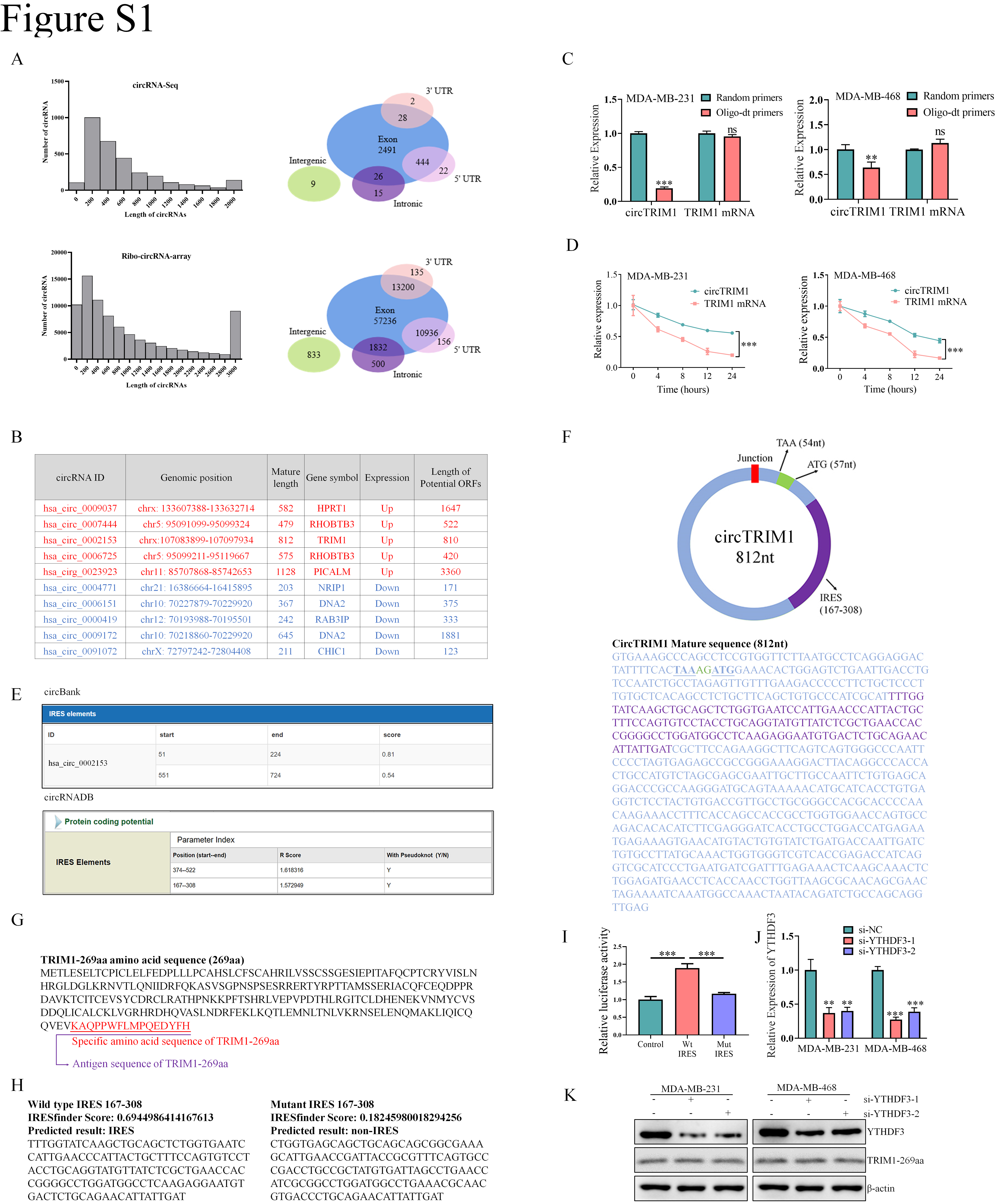

Supplement: Supplementary file 5 — Supplementary Material 5. [file 12943_2024_2019_MOESM5_ESM.jpg]

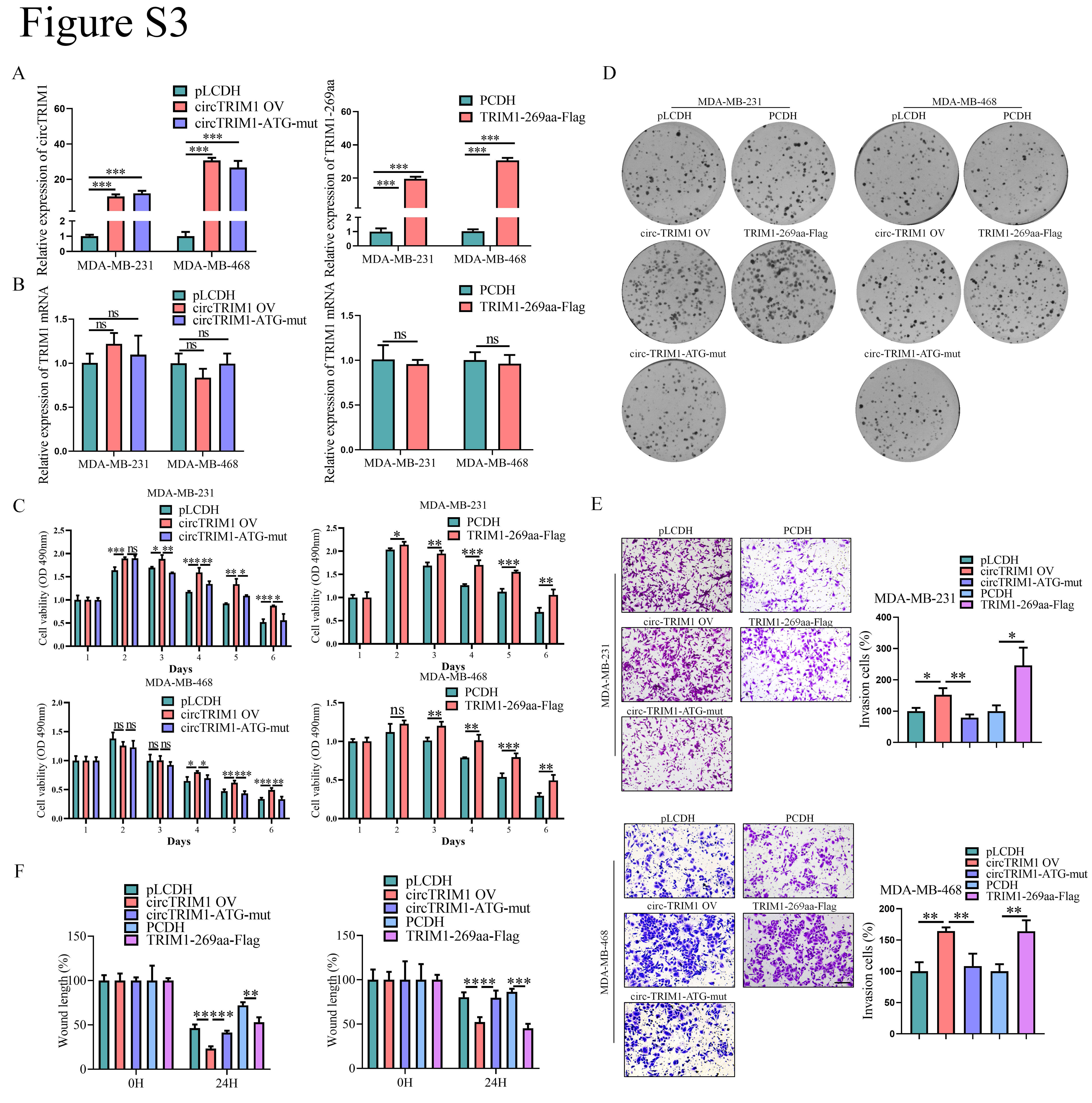

Supplement: Supplementary file 8 — Supplementary Material 8. [file 12943_2024_2019_MOESM8_ESM.jpg]
