# Supplementary material for: CircTRIM1 encodes TRIM1-269aa to promote chemoresistance and metastasis of TNBC via enhancing CaM-dependent MARCKS translocation and PI3K/AKT/mTOR activation
Source: Mol Cancer. 2024 May 16;23:102. doi: 10.1186/s12943-024-02019-6 (PMC11097450; doi:10.1186/s12943-024-02019-6)
Supplement: Supplementary file 2 — Supplementary Material 2: Figure S2. A. The position and sequence of circTRIM1 siRNAs. B. The efficiency of circTRIM1 siRNAs was measured by qRT‒PCR. C. The efficiency of circTRIM1 siRNAs was verified by FISH. Scale bars=40 μm. D. Effects of circTRIM1 knockdown on the protein expression of TRIM1-269aa. E. Effects of circTRIM1 knockdown on the expression of MID mRNA in TNBC cells. ns, nonsignificant; **P < 0.01; ***P < 0.001. [file 12943_2024_2019_MOESM2_ESM.docx]

**Table S.1. Primers used in this study**

| **Gene** | **Primer for PCR** | |
| --- | --- | --- |
|  | **Forward Primer** | **Reverse Primer** |
| circTRIM1/circTRIM1 Div | AGATGAACCTCACCAACCTG | CGGTGGTATTGTTGGGTA |
| circTRIM1 Con | CGCCAAGGGATGCAGTAAAA | TCGATCATTCAGGGATGCGA |
| TRIM1 mRNA | GCCAGTGTCTTGAACGGTCAAC | GAGAAGATGCAGTTGCCATAGCG |
| β-actin/β-actin Con | CACCATTGGCAATGAGCGGTTC | AGGTCTTTGCGGATGTCCACGT |
| β-actin Div | CATTGCTGACAGGATGCAGAAG | GGAAGGCTGGAAAAGAGCC |
| GAPDH | GTCTCCTCTGACTTCAACAGCG | ACCACCCTGTTGCTGTAGCCAA |
| U6 | CTCGCTTCGGCAGCACA | AACGCTTCACGAATTTGCGT |
| CALM2 | AGTGCTGCAGAACTTCGCCATG | CAAGGTCTTCACTTTGCTGTCATC |
