# Supplementary material for: CircTRIM1 encodes TRIM1-269aa to promote chemoresistance and metastasis of TNBC via enhancing CaM-dependent MARCKS translocation and PI3K/AKT/mTOR activation
Source: Mol Cancer. 2024 May 16;23:102. doi: 10.1186/s12943-024-02019-6 (PMC11097450; doi:10.1186/s12943-024-02019-6)
Supplement: Supplementary file 3 — Supplementary Material 3: Figure S3. A. Efficiency of the transfection of TNBC cells with circTRIM1 OV, circTRIM1-ATG-mut and TRIM1-269aa-Flag. B. Effects of the transfection of TNBC cells with circTRIM1 OV, circTRIM1-ATG-mut and TRIM1-269aa-Flag on the expression of TRIM1 mRNA. C. Time-dependent effects of circTRIM1, circTRIM1-ATG-mut and TRIM1-269aa on the chemoresistance of TNBC cells. D. Colony formation assays were used to evaluate the effects of circTRIM1 OV, circTRIM1-ATG-mut and TRIM1-269aa-Flag on the chemoresistance of TNBC cells. E. Effects of circTRIM1 OV, circTRIM1-ATG-mut and TRIM1-269aa-Flag on the invasion of TNBC cells. Scale bars = 200 μm. F. Effects of circTRIM1 OV, circTRIM1-ATG-mut and TRIM1-269aa-Flag on the migration abilities of TNBC cells based on a wound healing assay. *P< 0.05; **P < 0.01; ***P < 0.001. [file 12943_2024_2019_MOESM3_ESM.docx]

**Table S2. Antibodies used in the experiments.**

| **Antigen** | **Supplier** | **Catalog #** | **Application** |
| --- | --- | --- | --- |
| TRIM1-269aa | Abcepta | NA | IB (1:1000), IHC (1:200) |
| Flag | CST | 14793 | IB (1:1000) IP (1:50) IF (1:500) |
| Beclin1 | Proteintech | 11306-1-AP | IB (1:1000) |
| p62 | Proteintech | 18420-1-AP | IB (1:1000), IHC (1:500) |
| LC3B | Proteintech | 14600-1-AP | IB (1:1000) |
| β-actin | CST | 3700 | IB (1:1000) |
| MARCKS | Proteintech | 20661-1-AP | IB (1:1000), IP (1:50) |
| HA | CST | 3724 | IB (1:1000), IP (1:50) |
| p-MARCKS | CST | 8722 | IB (1:1000) |
| p-PDK1 | CST | 3438 | IB (1:1000) |
| PDK1 | CST | 5662 | IB (1:1000) |
| p-AKT | CST | 4060 | IB (1:1000) |
| AKT | CST | 9272 | IB (1:1000) |
| p-mTOR | Proteintech | 67778-1-Ig | IB (1:1000) |
| mTOR | CST | 2983 | IB (1:1000) |
| p-GSK3 | Abcam | ab68476 | IB (1:1000) |
| GSK3 | Abcam | ab185141 | IB (1:1000) |
| CALM2 | Beyotime | AG1288 | IB (1:1000) |
| PARP | CST | 9532 | IB (1:1000) |
| Caspase 3 | CST | 9662 | IB (1:1000) |
| Bax | Proteintech | 50599-2-Ig | IB (1:1000), IHC (1:500) |
| Bcl-2 | Proteintech | 12789-1-AP | IB (1:1000) |
| N-cadherin | Proteintech | 22018-1-AP | IB (1:1000), IHC (1:500) |
| E-cadherin | Proteintech | 20874-1-AP | IB (1:1000) |
| ZEB1 | Proteintech | 21544-1-AP | IB (1:1000) |
| Fibronectin | Proteintech | 15613-1-AP | IB (1:1000) |
| Vimentin | CST | 5741 | IB (1:1000) |
| Slug | CST | 9585 | IB (1:1000) |
| Snail | CST | 3879 | IB (1:1000) |
