# Supplementary material for: CircTRIM1 encodes TRIM1-269aa to promote chemoresistance and metastasis of TNBC via enhancing CaM-dependent MARCKS translocation and PI3K/AKT/mTOR activation
Source: Mol Cancer. 2024 May 16;23:102. doi: 10.1186/s12943-024-02019-6 (PMC11097450; doi:10.1186/s12943-024-02019-6)
Supplement: Supplementary file 6 — Supplementary Material 6. [file 12943_2024_2019_MOESM6_ESM.docx]

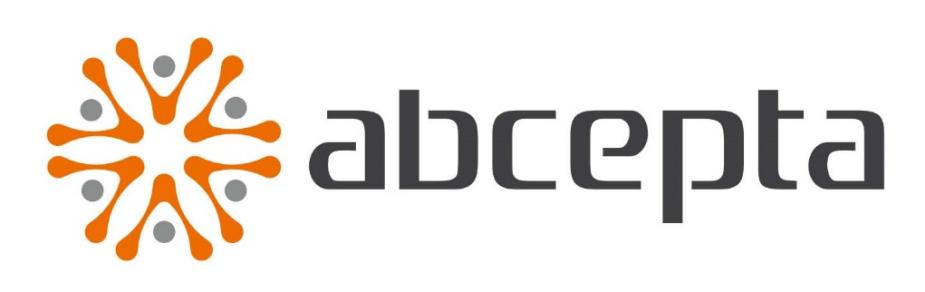


Project Progress Report for CS10071

Report Date: February 23, 2022

Abcepta Biotech Ltd. Co.

Room 301 #801, Building 1, No. 9 Shuangwei Road, Suzhou Industrial Park, China (Jiangsu) Pilot Free Trade Zone

[www.abcepta.com.cn](http://www.abcepta.com.cn)

Your Contact: Qinghua Yang

Email: tech-china@abcepta.com

Direct: 0512-88856768

The project has been conducted in accordance with sound scientific principles. The report accurately reflects the raw data from the experiments.

Report Prepared by Nannan Tian, Researcher, Custom Services Team, Abcepta

Report Reviewed by Xiaomin Liu, Manager, Project Management Team, Abcepta

Report Approved by Yushun Zhang, Director, R&D Team, Abcepta

**Summary**

The peptide synthesized by Abcepta was used to immunize 2 New Zealand Rabbits for polyclonal antibody production and purification. The produced anti sera were affinity purified using the immunizing peptide.

**Immunogens**

| **Project ID** | **Category** | **Antigen ID** | **Animal ID** |
| --- | --- | --- | --- |
| CS10071 | PolyclonalPolyclonalPolyclonal PolyclonalPolyclonal | sp211019CA | R03135-R03136 |

**Project Procedure**

**1. Immunization Protocol**

1) 1^st^ day: Pre-bleed 5ml.

2) 2^nd^ day: the first animal Immunization (200.0µg antigen; CFA)

3) 16^th^ day: the second animal Immunization (200.0µg antigen; IFA)

4) 30^th^ day: the third animal Immunization (100.0µg antigen; IFA)

5) 37^th^ day: 1st bleeding for ELISA validation.

6) 44^th^ day: the fourth animal Immunization (100.0µg antigen; IFA)

7) 51^st^ day: 2nd bleeding for ELISA validation.

8) 65^th^ day: 3th bleeding for ELISA validation.

9) If necessary, do further immunization and bleeding according to customer requirement.

10) Euthanasia animal, bleeding for Ab purification.

**2. Bleed/Immunization**

| **Action** | **Animal ID** | **Immunization/Bleed on** | **Amount** | **Comment** |
| --- | --- | --- | --- | --- |
| Immunization1 | R03135-R03136 | 2021/11/24 | 200.0µg | CFA |
| Immunization2 | R03135-R03136 | 2021/11/26 | 200.0µg | IFA |
| Immunization3 | R03135-R03136 | 2021/12/10 | 100.0µg | IFA |
| Bleeding1 | R03135-R03136 | 2021/12/24 | 10-15ml |  |
| Bleeding2 | R03135-R03136 | 2021/12/30 | 10-15ml |  |
| Immunization4 | R03135-R03136 | 2022/01/06 | 100.0µg | IFA |
| Bleeding3 | R03135-R03136 | 2022/01/07 | 10-15ml |  |
| Bleeding4 | R03135-R03136 | 2022/01/13 | 10-15ml |  |
| Immunization5 | R03135-R03136 | 2022/01/20 | 100.0µg | IFA |
| Bleeding5 | R03135-R03136 | 2022/01/21 | 10-15ml |  |
| Final bleeding | R03135-R03136 | 2022/01/27 | 30-50ml |  |

**3. Elisa Validation Protocol**

1. Dilute the antigen with coating buffer (0.01M PBS) to 1.25µg/ml and coat each well with 100µl this antigen solution, then incubate at 4°C overnight.
2. Wash the plate with PBST (0.06% tween-20) 3 times.
3. Block each well with 200µl of blocking buffer (2%BSA-PBS) and incubate at 37°C for 2 hours.
4. Wash the plate with PBST 3 times.
5. Add 100µl of serial diluted serum (1:1000, 1:2000, 1:4000 ,1:8000 and 1:16000) or primary antibody (1µg/ml, 0.5µg/ml, 0.25µg/ml, 0.125 µg/ml and 0.0625µg/ml) to each well of the plate and incubate at 37°C for 2 hours.
6. Wash the plate with PBST 5 times.
7. Add 100µl of pre-diluted HRP conjµgated secondary antibody to each well of the plate and incubate at 37°C for 1 hour.
8. Wash the plate with PBST 5 times.
9. Add 100µl of TMB to each well, and incubate at 37°C for 10 minutes in dark, then add stop solution.
10. Read the plate at 450 nm.

**4. Serum Elisa Data**

| **Rabbit ID** | **Antigen ID** | **Tested on** | **Bleed #** | **Blank** | **Pos 1:1000** | **Pos**  **1:2000** | **Pos 1:4000** | **Pos 1:8000** | **Pos 1:16000** |
| --- | --- | --- | --- | --- | --- | --- | --- | --- | --- |
| R03135 | sp211019CA | 2021/12/27 | 1 | 0.0874 | 3.5928 | 3.5282 | 3.4625 | 3.4391 | 3.0053 |
| R03136 | sp211019CA | 2021/12/27 | 1 | 0.0886 | 3.4221 | 3.3358 | 3.2138 | 3.0543 | 2.7231 |

*Note: Serum positive standard (1:4000 OD>1and 1:8000 OD>0.5 at 450nm)*

**5. Antibodies Purification Information**

| **Type** | **Animal ID** | **Antigen ID** | **Serum Vol.**  **(ml)** | **Lot No.** | **Ab Conc.**  **(mg/ml)** | **Ab Vol.**  **(ml)** | **Ab Mass (mg)** |
| --- | --- | --- | --- | --- | --- | --- | --- |
| Affinity Purification | R03135 | sp211019CA | 90 | SA220211X01 | 0.50 | 19.5 | 9.75 |
| Affinity Purification | R03136 | sp211019CA | 90 | SA220215X01 | 2.00 | 10 | 20.00 |

**6. Antibodies Elisa Data**

| **Rabbit ID** | **Antigen ID** | **Ab Lot#** | **Blank** | **Ab**  **1μg/ml** | **Ab**  **0.5μg/ml** | **Ab**  **0.25μg/ml** | **Ab**  **0.125μg/ml** | **Ab**  **0.0625μg/ml** |
| --- | --- | --- | --- | --- | --- | --- | --- | --- |
| R03135 | sp211019CA | SA220211X01 | 0.0937 | 3.6095 | 3.5766 | 3.5627 | 3.5556 | 3.4456 |
| R03136 | sp211019CA | SA220215X01 | 0.0983 | 3.5828 | 3.6167 | 3.5977 | 3.5434 | 3.4919 |

*Note: Antibody positive standard (0.125μg/ml OD>1 at 450nm)*

**Conclusion**

The ELISA data showed the antibody R03135（SA220211X01）and R03136（SA220215X01）is qualified.

Approved by

Yushun Zhang

Director, R&D Team, Abcepta
