# Supplementary material for: CircTRIM1 encodes TRIM1-269aa to promote chemoresistance and metastasis of TNBC via enhancing CaM-dependent MARCKS translocation and PI3K/AKT/mTOR activation
Source: Mol Cancer. 2024 May 16;23:102. doi: 10.1186/s12943-024-02019-6 (PMC11097450; doi:10.1186/s12943-024-02019-6)
Supplement: Supplementary file 7 — Supplementary Material 7. [file 12943_2024_2019_MOESM7_ESM.doc]

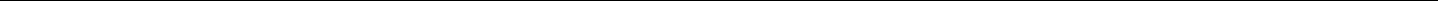


Sample Information

Product Name: sp211019CA
Sequence: CEVKAQPPWFLMPQEDYFH
Lot Number: P211104-M3845
Molecular Formula：C_111_H_153_N_25_O_29_S_2_

Moleclar Weight： 2365.68

Weight： 5mg*4

Store at： Cool dry place

Product Name ：sp211019CA

Sequence ：CEVKAQPPWFLMPQEDYFH

Instrument No ：N/A

Lot.No ：P211104-M3845

Column ：Kromasil, 100-5-C18,(4.6mm i.d.,250 mm L)

Solvent A ：0.1% TFA in Acetonitrile

Solvent B ：0.1% TFA in H2O

Gradient ：

|  | A | B |
| --- | --- | --- |
| 0.01min | 21 | 79 |
| 25min | 46 | 54 |
| 30min | 100 | 0 |
| 31min | 21 | 79 |
| 31.1min | stop |  |

Flow rate : 1.0ml/min

Wavelength : 220nm

Volume :10 µl


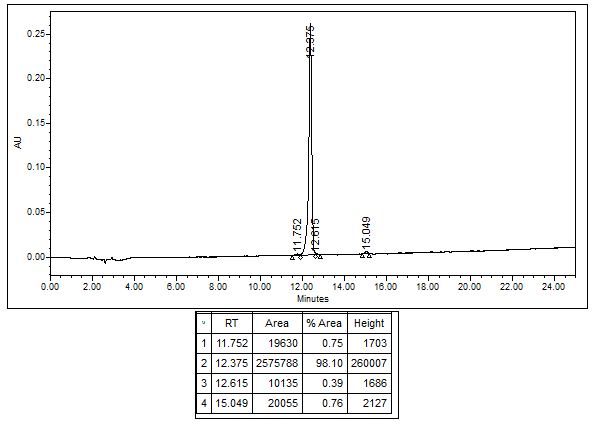


|  |
| --- |

# Mass Spectrometry Report

#

|  |  | |  | | |  | | | |
| --- | --- | --- | --- | --- | --- | --- | --- | --- | --- |
| Dissolution method | | :0.1%HCOOH+ACN | | | Interface | | :ESI | Prerod Bias | :+3.5kv |
| Date Acquired | | :2021////12121211221111103/26 | | 11/05 4:22:5103 | Nebulizing Gas Flow | | :500L/hr | Detector | :-0.2kv |
| Injection Volume | | :2.5ul | | | CDL Temp | | :400C | T.Flow | :0.35ml/min |
| Block Temp | | :150 | | | CDL Volt | | :+15v | B.conc | :50%H2O/50%MEOH |
